# Sonosensitive capsules for brain thrombolysis increase ischemic damage in a stroke model

**DOI:** 10.1186/s12951-022-01252-9

**Published:** 2022-01-21

**Authors:** Clara Correa-Paz, María F. Navarro Poupard, Ester Polo, Manuel Rodríguez-Pérez, Martina Migliavacca, Ramón Iglesias-Rey, Alberto Ouro, Elena Maqueda, Pablo Hervella, Tomás Sobrino, José Castillo, Pablo del Pino, Beatriz Pelaz, Francisco Campos

**Affiliations:** 1 Clinical Neurosciences Research Laboratory (LINC), Health Research Institute of Santiago de Compostela (IDIS), 15706 Santiago de Compostela, Spain; 2 Center for Research in Biological Chemistry and Molecular Materials (CiQUS), University of Santiago de Compostela, 15782 Santiago, Spain; 3 Department of Biochemistry and Molecular Biology, University of Santiago de Compostela, 15782 Santiago de Compostela, Spain; 4 Department of Particle Physics, University of Santiago de Compostela, 15782 Santiago de Compostela, Spain; 5 Department of Inorganic Chemistry, University of Santiago de Compostela, 15782 Santiago de Compostela, Spain

**Keywords:** Capsules, Ischemic stroke, Layer-by-layer, Magnetic resonance imaging, Tissue plasminogen activator, Ultrasound

## Abstract

**Background:**

Ischemic stroke is the most common cerebrovascular disease and is caused by interruption of blood supply to the brain. To date, recombinant tissue plasminogen activator (rtPA) has been the main pharmacological treatment in the acute phase. However, this treatment has some drawbacks, such as a short half-life, low reperfusion rate, risk of hemorrhagic transformations, and neurotoxic effects. To overcome the limitations of rtPA and improve its effectiveness, we recently designed sonosensitive sub-micrometric capsules (SCs) loaded with rtPA with a size of approximately 600 nm, synthesized using the layer-by-layer (LbL) technique, and coated with gelatine for clot targeting. In this study, we evaluated the rtPA release of ultrasound (US)-responsive SCs in healthy mice and the therapeutic effect in a thromboembolic stroke model.

**Results:**

In healthy mice, SCs loaded with rtPA 1 mg/kg responded properly to external US exposure, extending the half-life of the drug in the blood stream more than the group treated with free rtPA solution. The gelatine coating also contributed to stabilizing the encapsulation and maintaining the response to US. When the same particles were administered in the stroke model, these SCs appeared to aggregate in the ischemic brain region, probably generating secondary embolisms and limiting the thrombolytic effect of rtPA. Despite the promising results of these thrombolytic particles, at least under the dose and size conditions used in this study, the administration of these capsules represents a risk factor for stroke.

**Conclusions:**

This is the first study to report the aggregation risk of a drug carrier in neurological pathologies such as stroke. Biocompatibility analysis related to the use of nano-and microparticles should be deeply studied to anticipate the limitations and orientate the design of new nanoparticles for translation to humans.

**Graphical Abstract:**

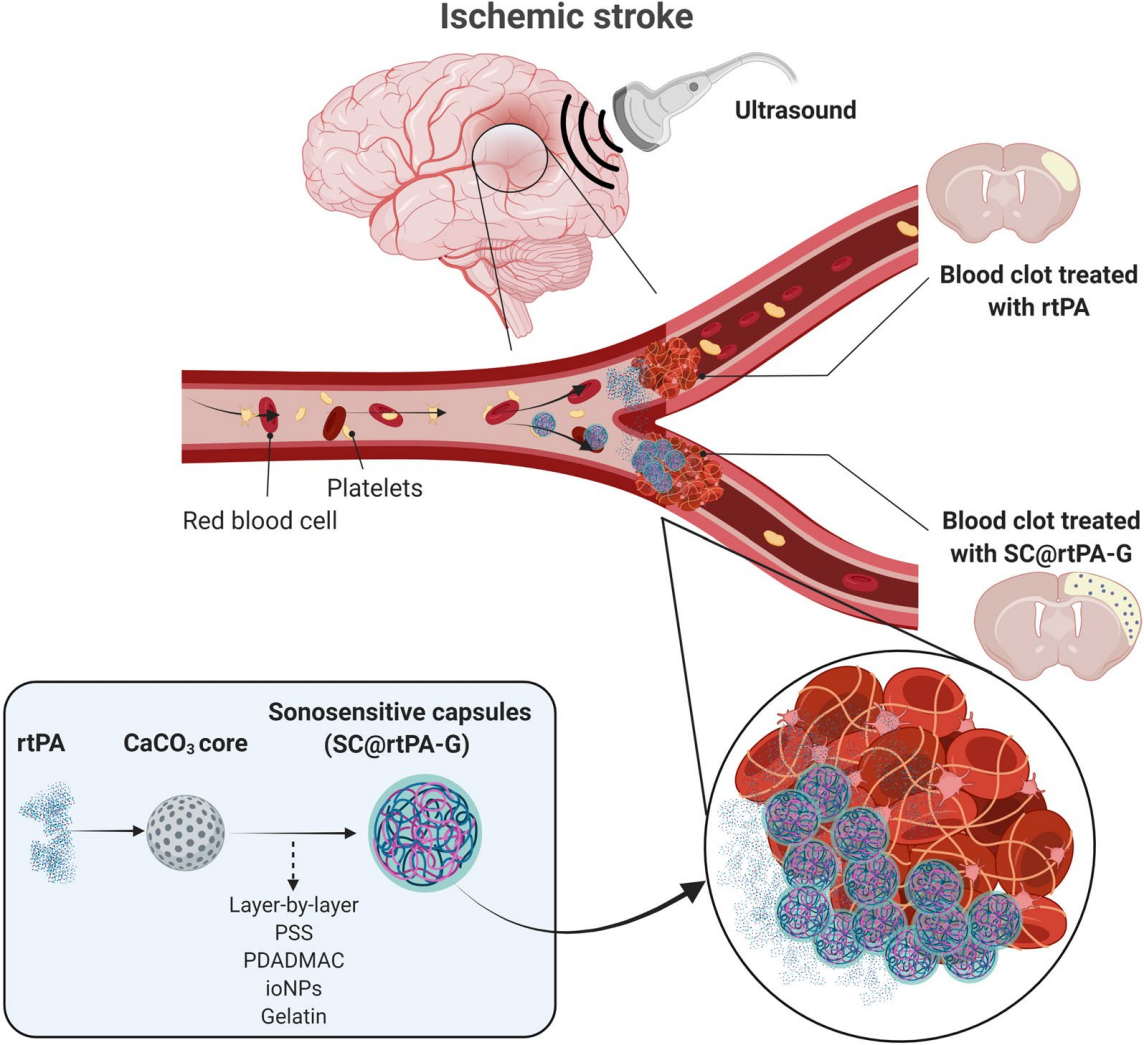

**Supplementary Information:**

The online version contains supplementary material available at 10.1186/s12951-022-01252-9.

## Background

Stroke is a cerebrovascular disease and one of the main causes of death and disability in developed countries. It occurs as a result of disruption of cerebral blood flow (CBF), which induces metabolic and cellular changes, leading to cell death and alteration of brain function. Ischemic stroke is caused by an obstruction in the vessel, often due to the presence of a clot, and constitutes approximately 85% of all stroke events. Protective interventions focused on the prevention of cell death after an ischemic event have not yet demonstrated benefits in clinical practice, and recovery therapies are still far from becoming a treatment strategy for stroke patients [1, 2].

Reperfusion therapies, based on the use of pharmacological thrombolysis and/or mechanical recanalization, focus on restoring CBF in the first hours after stroke onset [3]. Pharmacological thrombolysis with intravenous (IV) recombinant tissue plasminogen activator (rtPA) is the main approved drug treatment for patients with acute ischemic stroke. However, its use has some limitations, such as a low reperfusion rate, which occurs in—40–50% of treated patients, the risk of hemorrhagic transformation in 7–15% of cases, and neurotoxic effects [4–6]. These drawbacks limit the rtPA therapeutic window to 4.5 h after symptom onset [3]. Furthermore, the short half-life of rtPA (~ 5 min) requires that it be administered as an initial loading bolus (10% of the dose) followed by a continuous infusion for 1 h (90% of the dose) [7, 8].

Nanotechnology has emerged as a promising strategy for overcoming the limitations of rtPA and improving its efficacy. These nanomedical approaches focus on targeting the drug to the arterial clot-region and inducing a controlled drug release, with a final goal of increasing the number of patients that can safely benefit from rtPA [9].

Due to the limitations involving the encapsulation of a large (~ 70 kDa) and sensitive enzyme, such as rtPA, specific nanomedical carriers have been developed to encapsulate and optimize the efficacy and safety of rtPA for stroke, such as liposomes, polymeric nanoparticles, magnetic nanoparticles, microbubbles, and echogenic liposomes, all with different levels of success [9]. For many of these approaches, the most common in vivo model used to evaluate therapeutic efficacy has been a thromboembolic stroke model induced by electrocoagulation or FeCl_3_ application on the cerebral artery. However, these models are characterized by the generation of a platelet-rich thrombus when rtPA-mediated thrombolysis is mainly active on fibrin clots [10–13]. For example, the rates of early arterial recanalization after rtPA administration in platelet-rich thrombi is ~ 6% while ~ 30%, in the fibrin thrombi [14, 15]. Therefore, selection of a more rtPA-sensitive model is convenient for evaluating thrombolytic therapies.

In recent years, sonosensitive sub-micrometric capsules synthesized by the layer-by-layer (LbL) technique have been described as a promising strategy for drug delivery for many reasons: (i) versatility of polymeric composition; (ii) loading capabilities of sensitive molecules of different sizes, from small molecules (e.g., dyes) to large macromolecules (proteins, enzymes, oligonucleotides, etc.); (iii) mild conditions for core removal, which ensures the activity of bioactive molecules; and (iv) stabilization and protection from natural inhibitors of the cargo [16–18]. Therefore, these properties convert LbL-based particles into promising carriers for rtPA release. However, despite the opportunities offered by this nanotechnology, its biocompatibility and in vivo drug delivery efficacy are poorly explored. Any advances in this field could pave the way for further development of carriers for large molecules (≥ 10 kDa) [16].

For this purpose, we recently designed sonosensitive SCs loaded with rtPA with a size of approximately 600 nm and synthesized by the LbL technique (hereafter referred to as nanocapsules, SCs) for subsequent ultrasound (US)-controlled delivery [19]. Based on the affinity of gelatine for von Willebrand factor (VWF) [20], a thrombus component, it was used to coat and target the SCs to the arterial occluded region. We demonstrated that rtPA encapsulation in these microsystems prevents its endogenous biological inactivation, does not interfere with thrombolytic activity, and promotes the breakdown of blood clots after US application in in vitro assays. Finally, in vivo studies in healthy animals confirmed the release of rtPA from our capsules, which were intravenously administered to mice under external US triggering [19].

In the present study, the biocompatibility and therapeutic efficacy of our newly designed thrombolytic carriers in ischemic stroke conditions was tested for the first time, using a fibrin-rich thromboembolic stroke model. This model, induced by an in situ injection of thrombin in the middle cerebral artery (MCA), appears to be pathophysiologically closer to human stroke, and is widely accepted to evaluate the recanalization effects of rtPA [21, 22].

We validated that our SCs responded to external US exposure by releasing rtPA and that the gelatine coating helps to stabilize the encapsulation and to maintain the response to US in vivo. However, when the therapeutic effect of these SCs was evaluated in the selected stroke model, we observed, by in vivo magnetic resonance imaging (MRI), that these SCs tended to aggregate in the ischemic region, leading to an increase in infarct size, independent of the gelatine coating or US application.

## Results

### Synthesis and characterization of capsules

**Fig. 1:**
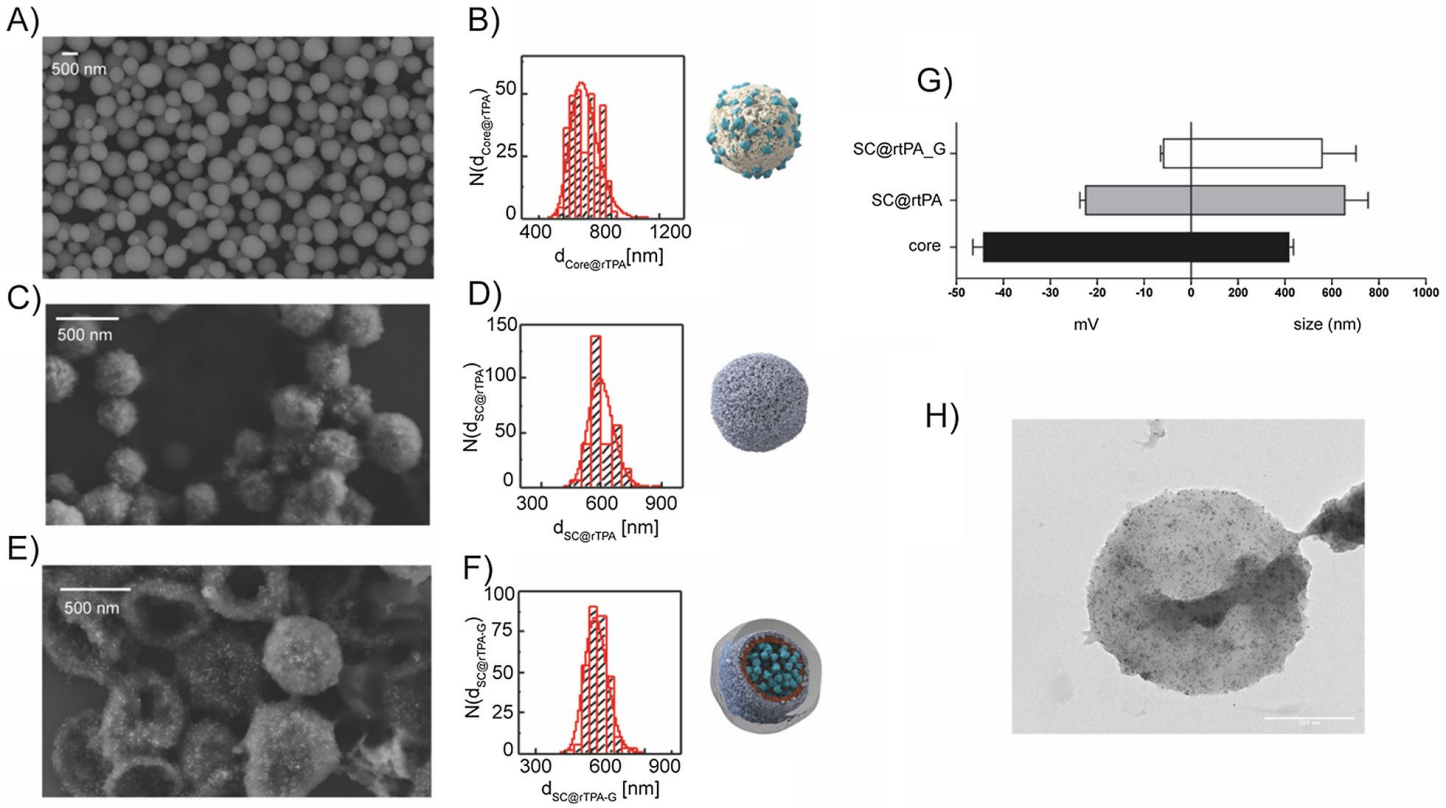
**A** Scanning electron microscope (SEM) micrograph of cores (scale bar is 500 nm). **B** Size histogram (d_core@rtPA_: mean diameter of core@rtPA, rtPA loaded vaterite CaCO_3_ cores) obtained after measuring the diameter of 300 core@rtPA using ImageJ; d_core@rtPA_ = 580 ± 111 nm. **C** SEM micrograph of SC@rtPA (no gelatine) before core removal (scale bar is 500 nm). **D** Size histogram (d_SC@rtPA_: mean diameter of SC@rtPA) obtained after measuring the diameter of over 300 SC@rtPA using ImageJ; d_SC@rtPA_ = 599 ± 60 nm. **E** SEM micrograph of SC@rtPA-G (gelatine) after core removal (scale bar is 500 nm). **F** Size histogram (d_SC@rtPA-G_: mean diameter of SC@rtPA-G) obtained after measuring the diameter of over 300 SC@rtPA-G using ImageJ; d_SC@rtPA-G_ = 632 ± 65 nm. **G** Mean average hydrodynamic diameters and ζ-potential values for core@rtPA, SC@rtPA and SC@rtPA-G. **H** Transmission electron microscopy (TEM) micrograph of SC@rtPA after core removal (scale bar is 500 nm); ioNPs are distinguishable as dark dots in the polymer shell of collapsed SCs

Different batches of SCs loaded with rtPA, with or without gelatine, were synthesized following the same synthesis protocol described in our previous study [19]. SCs with gelatine (G) are referred to as SC@rtPA-G and SCs without gelatine are referred to as SC@rtPA. Monodispersed SCs were synthesized with a spherical morphology and diameter with a particle size of ~ 600 nm (in diameter), as determined by electron microscopy (Fig. 1A–F; Additional file 1: Figs. S1, S2). The colloidal stability of all synthesized SCs was confirmed after the layer-by-layer process, core removal, and gelatine coating, as indicated by dynamic light scattering (DLS) analysis (Fig. 1G; Additional file 1: Fig. S3, Table S1). In both types of SCs produced, SC@rtPA and SC@rtPA-G, the concentration of rtPA, concentration of SCs, and pg of rtPA/SCs were analyzed in order to use a therapeutic dose of rtPA (1 mg/kg) and a similar number of SCs for the animal treatment (see Table 1). Furthermore, iron oxide nanoparticles (ioNPs) were added to make these SCs suitable for MRI imaging and inductively coupled plasma mass spectrometry (ICP-MS) analysis (Fig. 1H). Particle synthesis and protein loading (Additional file 1: Figs. S4, S5) did not interfere with the stability and thrombolytic activity of the enzyme, as previously demonstrated [19]. Moreover, encapsulated rtPA was protected from its natural inhibitor, plasminogen activator inhibitor (PAI-1).

**Table 1:** Summary characterization of different batches of nanocapsules before animal administration

SC	rtPA concentration (mg/mL)	Number of nanocapsules (SC/mL)	pg rtPA/SC
SC@rtPA	0.07	3.81 × 10^9^	0.02
	0.07	3.87 × 10^9^	0.02
	0.09	5.99 × 10^9^	0.02
SC@rtPA-G	0.10	2.58 × 10^9^	0.04
	0.13	4.49 × 10^9^	0.03
	0.10	1.97 × 10^9^	0.05

### In vivo release of the rtPA from SCs with and without gelatine by US application

The in vivo triggering of rtPA release from SCs was initially evaluated in healthy animals. The following experimental groups were included in the analysis: (1) control or vehicle group (treated with saline), (2) free solution of rtPA (1 mg/kg, bolus administration), (3) SC@rtPA (1 mg/kg rtPA, bolus administration), and (4) SC@rtPA-G (1 mg/kg rtPA, bolus administration). The four groups were combined with or without US application. In clinical practice, for the treatment of acute ischemic stroke, rtPA is administered as 0.9–1 mg/kg, 10% of volume as loading bolus and followed by 90% as infusion (see prescribing information of Alteplase^®^). However, in preclinical mouse models, since the fibrinolytic system is ten-fold less sensitive to rtPA than the human system, most preclinical studies were performed with 10 mg/kg instead of 0.9 mg/kg rtPA [23, 24]. To compare the response of SCs to the clinical protocol, an additional group treated with an infusion of 10 mg/kg rtPA (rtPAi) was included in the study. This last group was not combined with US application.

**Fig. 2:**
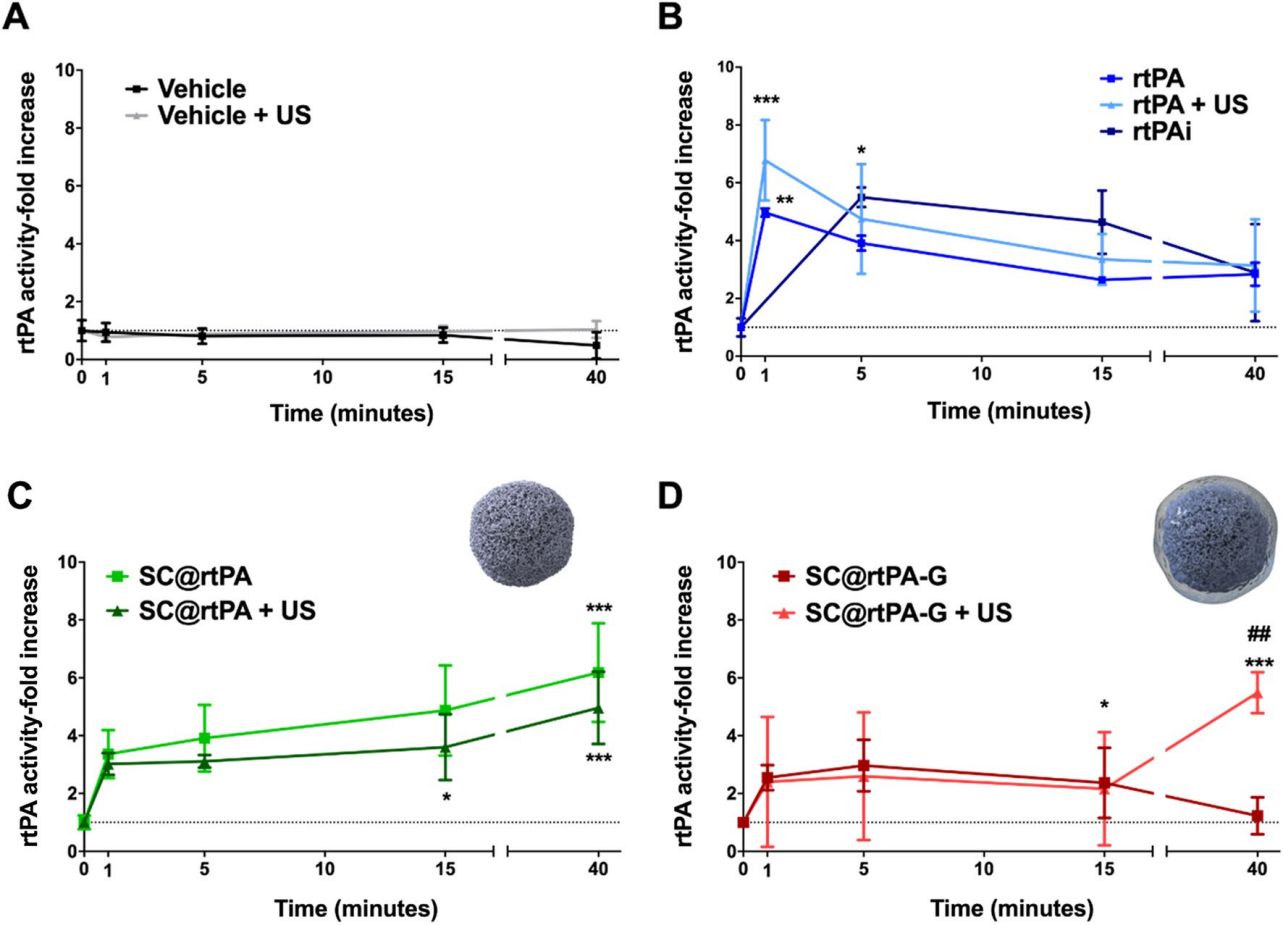
rtPA plasmatic profile of the groups of healthy animals treated with **A** vehicle (saline) and vehicle with ultrasound (US) administration; **B** rtPA (1 mg/kg as bolus), rtPA (1 mg/kg as bolus) with US and rtPAi (10 mg/kg administered 10% as bolus and 90% as infusion); **C** SCs@rtPA (1 mg/kg as bolus) and SCs@rtPA (1 mg/kg as bolus) with US; **D** SCs@rtPA-G (1 mg/kg as bolus) and SCs@rtPA-G (1 mg/kg as bolus) with US. The dotted lines represent the plasma activity of the control group treated with the vehicle. The square symbols represent the groups treated without US, while the triangles are the groups treated with US. All data are represented as mean ± SD (n = 3 per treatment group). In all data statistical analysis was assessed by a one-way ANOVA followed by post-hoc Brown-Forsythe evaluation: *(P < 0.05), **(P < 0.01), ***(P < 0.001) compared to vehicle, and, ^##^(P < 0.01) compared to SC@rtPA-G at the same timepoints

As shown in Fig. 2A, treatment with vehicle or US did not affect rtPA plasma activity. According to the fast blood metabolization of the enzyme, bolus administration of non-encapsulated rtPA (1 mg/kg) showed an increase in thrombolytic activity, which was immediately reduced 5 min later. As expected, infusion of 10 mg/kg rtPA showed a sustained increase for at least 15 min during administration, according to the administration protocol (10% bolus + 90% infusion over 40 min). The combination of US and non-encapsulated rtPA (1 mg/kg, bolus administration) did not show any remarkable variation in rtPA activity (Fig. 2B). Administration of SCs@rtPA (1 mg/kg) caused a progressive increase in rtPA plasma activity during the 40 min of follow-up. In addition, rtPA release was not affected by US application during the 40 min period of the study (Fig. 2C). The sustained rtPA release observed for SC@rtPA with and without US might be the result of the in vivo degradation processes of SCs. In the case of SCs@rtPA-G (1 mg/kg), a progressive increase in rtPA plasma activity was also observed, although this increase was lower than that observed in the SCs@rtPA group. US application (over 40 min) after SCs@rtPA-G administration triggered rtPA release, reaching an approximately six-fold increase with respect to basal levels (Fig. 2D). Comparing the US sensitivity of SC@rtPA-G and SC@rtPA, it seems that the presence of gelatine enhances the in vivo stability of the nanoformulation, thereby promoting its responsiveness to US.

### Ultrasound safety study in healthy and ischemic animals

**Fig. 3:**
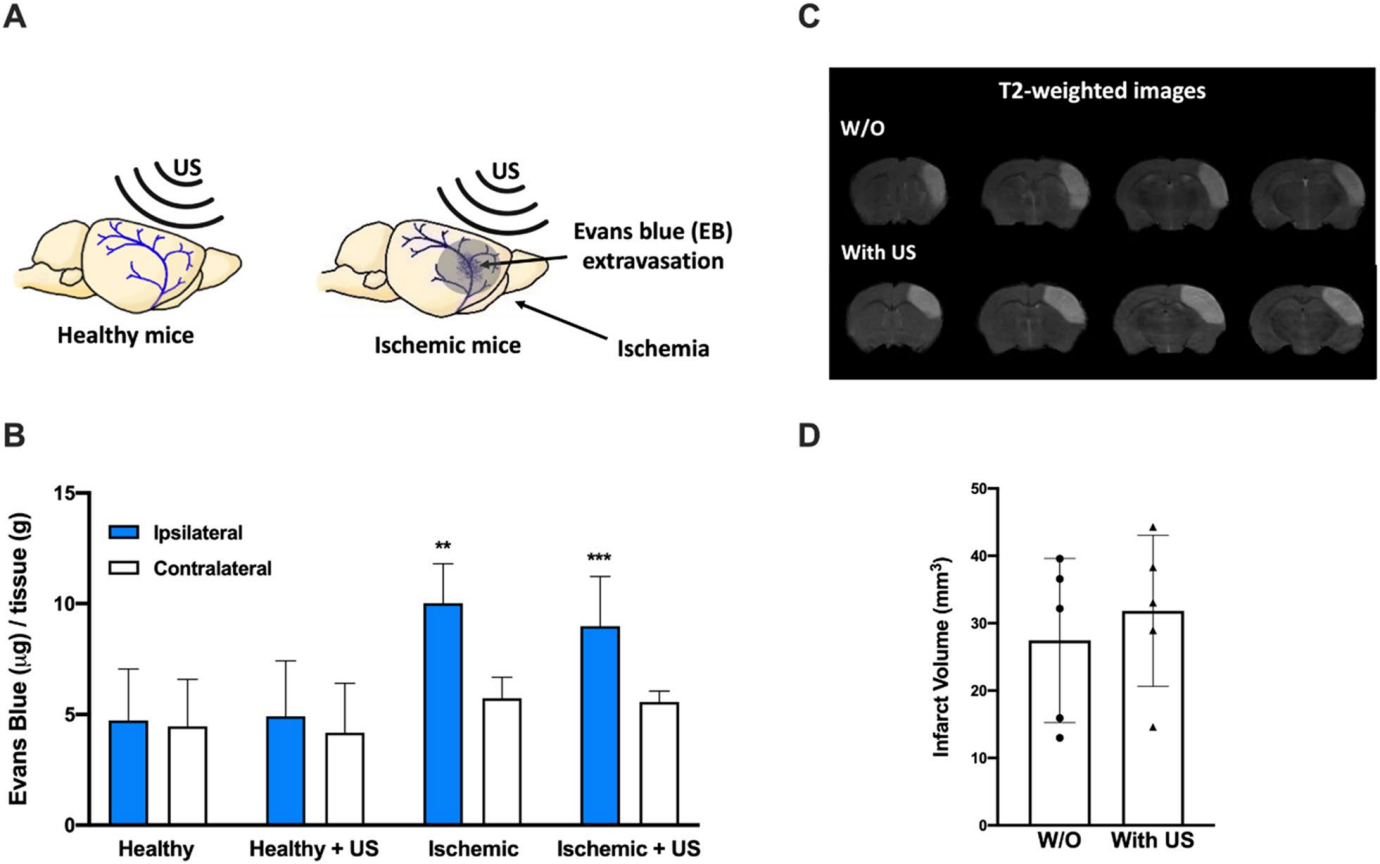
**A** Schematic representation of EB extravasation after ischemia. When the blood brain barrier (BBB) is compromised, the extravasation of EB into the parenchyma occurs. **B** Analysis of the BBB disruption in healthy and ischemic mice under ultrasound (US) exposure determined by EB extravasation. Amount (µg) of EB was corrected by the brain tissue weight (g). **C** MRI of the ischemic animals with and without (W/O) US. **D** Ischemic volume analysis with and without US application. All data are represented as mean ± SD (n = 5 per treatment group). In all data statistical significance was assessed by the *t*-test. **P < 0.01; *** *P* < 0.001. Each ipsilateral hemisphere was compared with its respective contralateral hemisphere

Safety analyses performed in an independent group of healthy and ischemic animals demonstrated that external US (0.72 W/cm^2^, 2 MHz) for 40 min in the MCA territory did not damage the blood–brain barrier (BBB) integrity determined by Evans Blue (EB) extravasation (Fig. 3A, B) or increased ischemic damage (Fig. 3C, D). An increase in EB extravasation was observed in the ipsilateral hemisphere of ischemic mice due to infarct lesions (Fig. 3A, B). However, there were no differences in EB extravasation (related to BBB damage) in ischemic lesions between the ischemic animals treated with and without US.

### Arterial reperfusion rate of the microsystems in the ischemic model

**Fig. 4:**
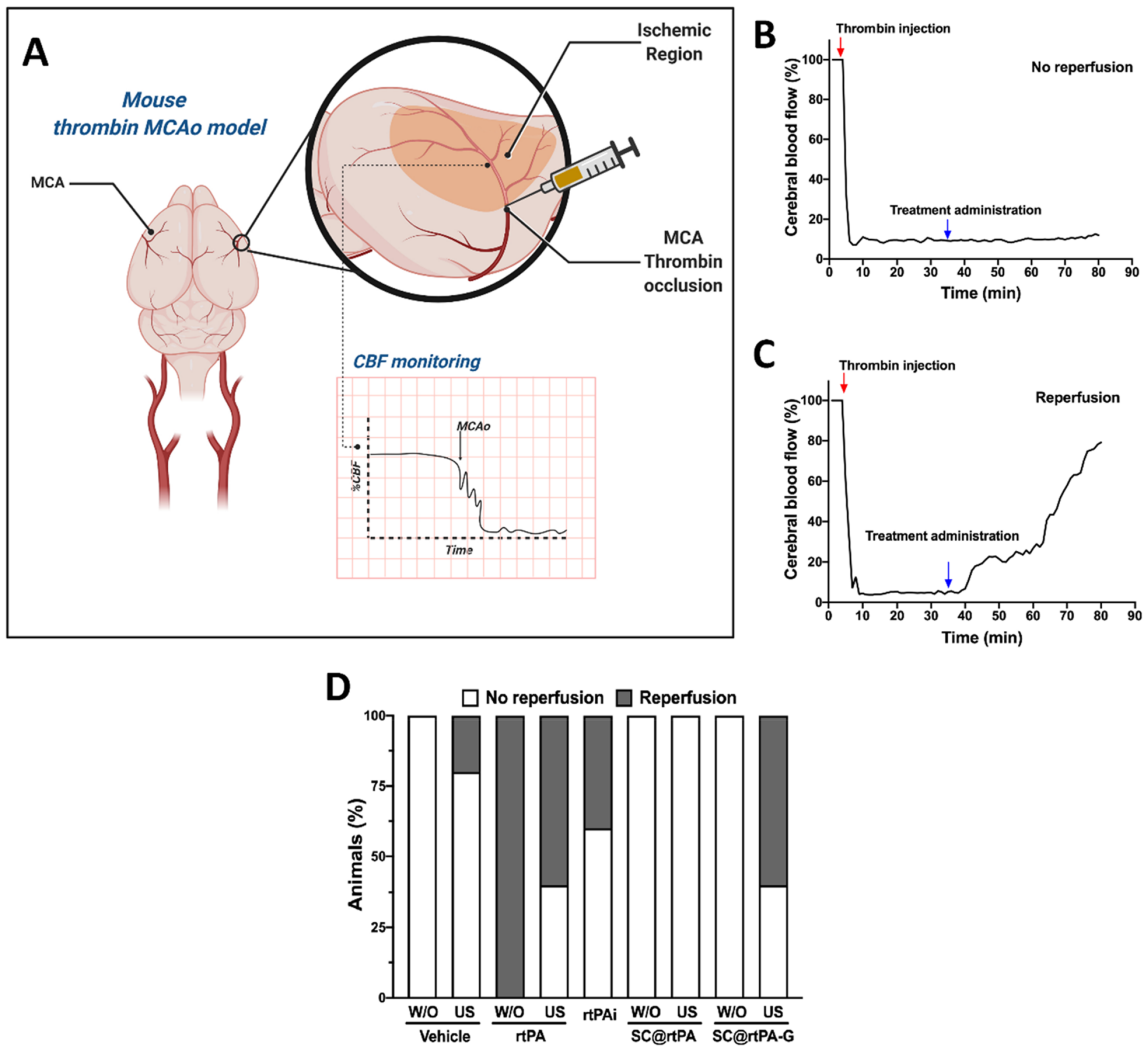
**A** Murine model of thromboembolic stroke induced by the occlusion of the medial cerebral artery (MCAo) with thrombin injection. **B** Representation of the Cerebral Blood Flow (CBF) recording without reperfusion and **C** with reperfusion. Red arrows indicate the arterial occlusion induced by thrombin injection, and 35 min later the treatment administration (blue arrows): saline or vehicle (vehicle, vehicle with ultrasound), rtPA free solution (rtPA (1 mg/kg as bolus), rtPA with US (1 mg/kg as bolus), rtPAi (rtPA 10 mg/kg as bolus and infusion), SC@rtPA (1 mg/kg as bolus), SC@rtPA with US (1 mg/kg as bolus), SC@rtPA-G (1 mg/kg as bolus) and SC@rtPA-G with US (1 mg/kg as bolus). **D** Reperfusion rate in the different groups (n = 5 per treatment group) determined by laser Doppler monitoring. Successful reperfusion was considered when at least 40% of the basal CBF was recovered. Animals treated without ultrasound (US) are marked as W/O

Once the* in vivo* release profile of rtPA from SCs was evaluated, the same treatments were tested in a thromboembolic ischemic model induced by in situ injection of thrombin in the MCA (Fig. 4A). In all groups, local injection of thrombin in the MCA caused an immediate drop (to ~ 80% of baseline) in CBF (Fig. 4B). Thirty minutes after the artery occlusion, different experimental treatments were administered. As was previously reported using the same model [25], arterial reperfusion was considered when at least 40% of the basal CBF was recovered within the first 40 min after treatment administration (Fig. 4C).

Saline treatment, which was used as a vehicle for the control group, did not affect thrombin arterial occlusion during CBF monitoring. In line with the clinical data [4] and a previous meta-analysis using the same stroke model [22], complete or partial arterial reperfusion was observed in the three groups treated with a free solution of non-encapsulated rtPA (both 1 mg/kg as bolus administration with and without US and 10 mg/kg as infusion). In the four groups treated with SCs, only the SC@rtPA-G group with US showed partial reperfusion, as expected with the controlled sustained rtPA-release induced by the US treatment. However, in the other three SC groups, SC@rtPA-G without US and SC@rtPA with and without US, none of the animals treated showed successful reperfusion (Fig. 4D).

### Effect of the SCs on infarct volume

**Fig. 5:**
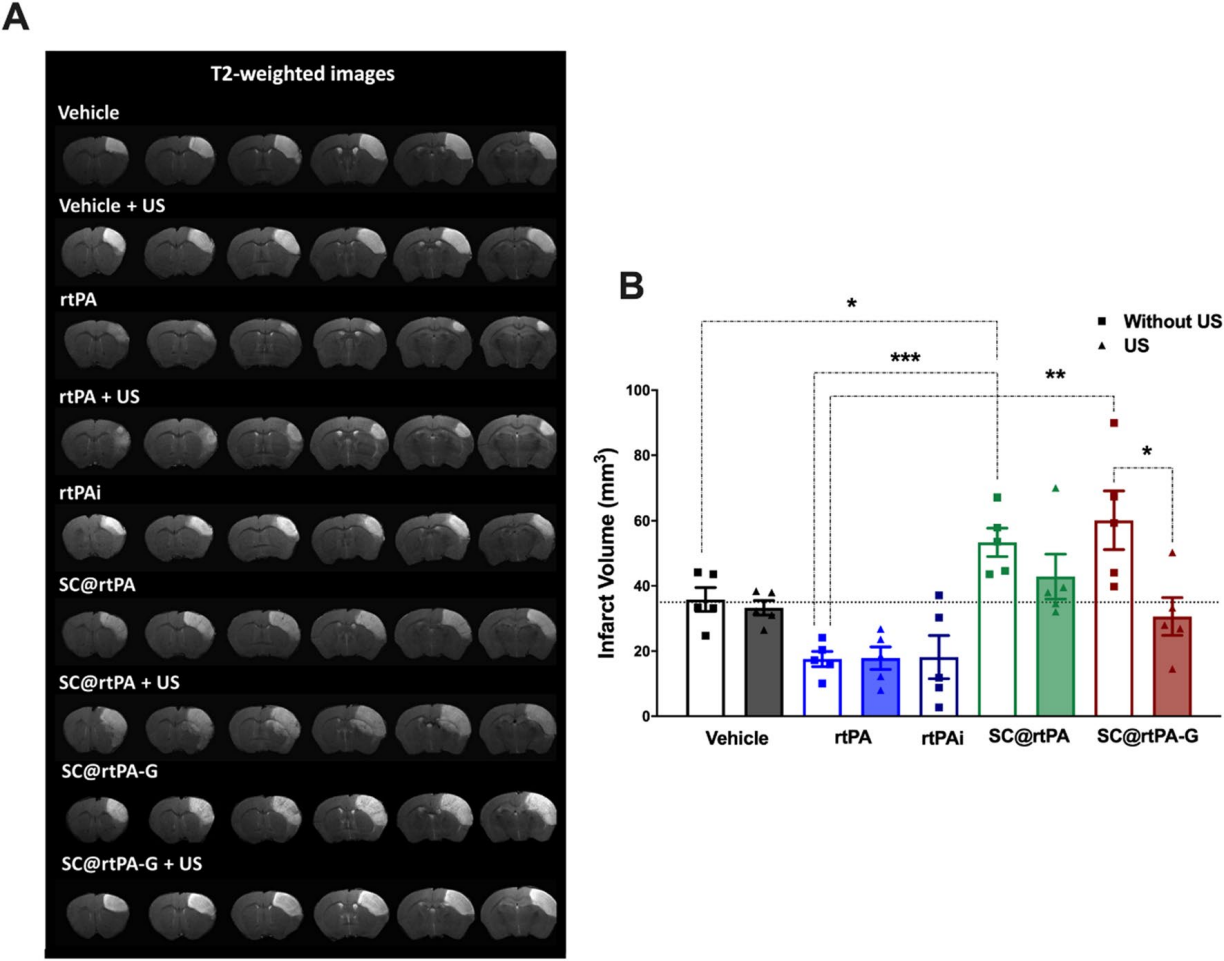
**A** Ischemic lesion (white brain region) determined by T2-weighted at 24 h after the treatment administration in the 9 experimental groups: vehicle, vehicle with ultrasound (US), rtPA (1 mg/kg as bolus), rtPA with US (1 mg/kg as bolus), rtPAi (rtPA 10 mg/kg as bolus and infusion), SC@rtPA (1 mg/kg as bolus), SC@rtPA with US (1 mg/kg as bolus), SC@rtPA-G (1 mg/kg as bolus) and SC@rtPA-G with US (1 mg/kg as bolus). **B** Analysis of the infarct volume at 24 h after treatment administration. The dotted lines represent the median of the vehicle group. The square symbols and empty columns represent the groups treated without ultrasound, while the triangles and colored columns are the groups treated with ultrasound. All data are represented as mean ± SD (n = 5 per group of treatment). In all data statistical analysis was assessed by the one-way ANOVA followed by post-hoc Brown-Forsythe test. *(P < 0.05), ^**^(P < 0.01); ^***^(P < 0.001)

The correlation between the reperfusion rate and ischemic infarct lesion was subsequently evaluated in the same animals by MRI at 24 h (Fig. 5A) and 3 days (Additional file 1: Fig. S6). MRI scan showed that animals, with permanent arterial occlusion, treated with the vehicle, presented an average infarct volume of 35 mm^3^ 24 h after ischemic induction. Thrombolytic recanalization with non-encapsulated rtPA (1 mg/kg as bolus, 1 mg/kg as bolus and US, or 10 mg/kg as infusion) produced a significant reduction in infarct volume compared with the control group (average of 18 mm^3^ vs*.* 35 mm^3^ at 24 h) (Fig. 5B). The same infarct volume (~ 18 mm^3^) confirmed that the recanalization efficacy was very similar in these three groups treated with free solution of rtPA, thus, neither US or the protocol administration interferes with drug activity.

These findings are clinically relevant, as they show that the rtPA infusion protocol is not necessarily required to achieve a beneficial effect. In addition, although we used 10 mg/kg rtPA, as is recommended for rodents, a dose as low as 1 mg/kg is sufficient to produce successful recanalization. Therefore, our data supports previous evidence [23] that the human clinical dose of 0.9–1 mg/kg rtPA is as appropriate as that of 10 mg/kg for preclinical stroke studies in rodents.

On the other hand, analysis of ischemic lesions in animals treated with SCs@rtPA, SCs@rtPA + US, and SCs@rtPA-G showed a higher infarct volume compared to the control group with permanent arterial occlusion (53 mm^3^, 43 mm^3^, and 60 mm^3^, respectively, vs*.* 35 mm^3^, which is an increment of approximately 1.5, 1.2 and 1.7 times, respectively). Only the group treated with SC@rtPA-G and US, with partial recanalization, showed a lower infarct, but without a therapeutic effect, as the infarct size was similar to that of the vehicle group (31 mm^3^ *vs.* 35 mm^3^). Infarct volumes determined 3 days after ischemia showed the same trend observed at 24 h, as indicated in Additional file 1: Fig. S6.

### Fate and distribution of the SCs

**Fig. 6:**
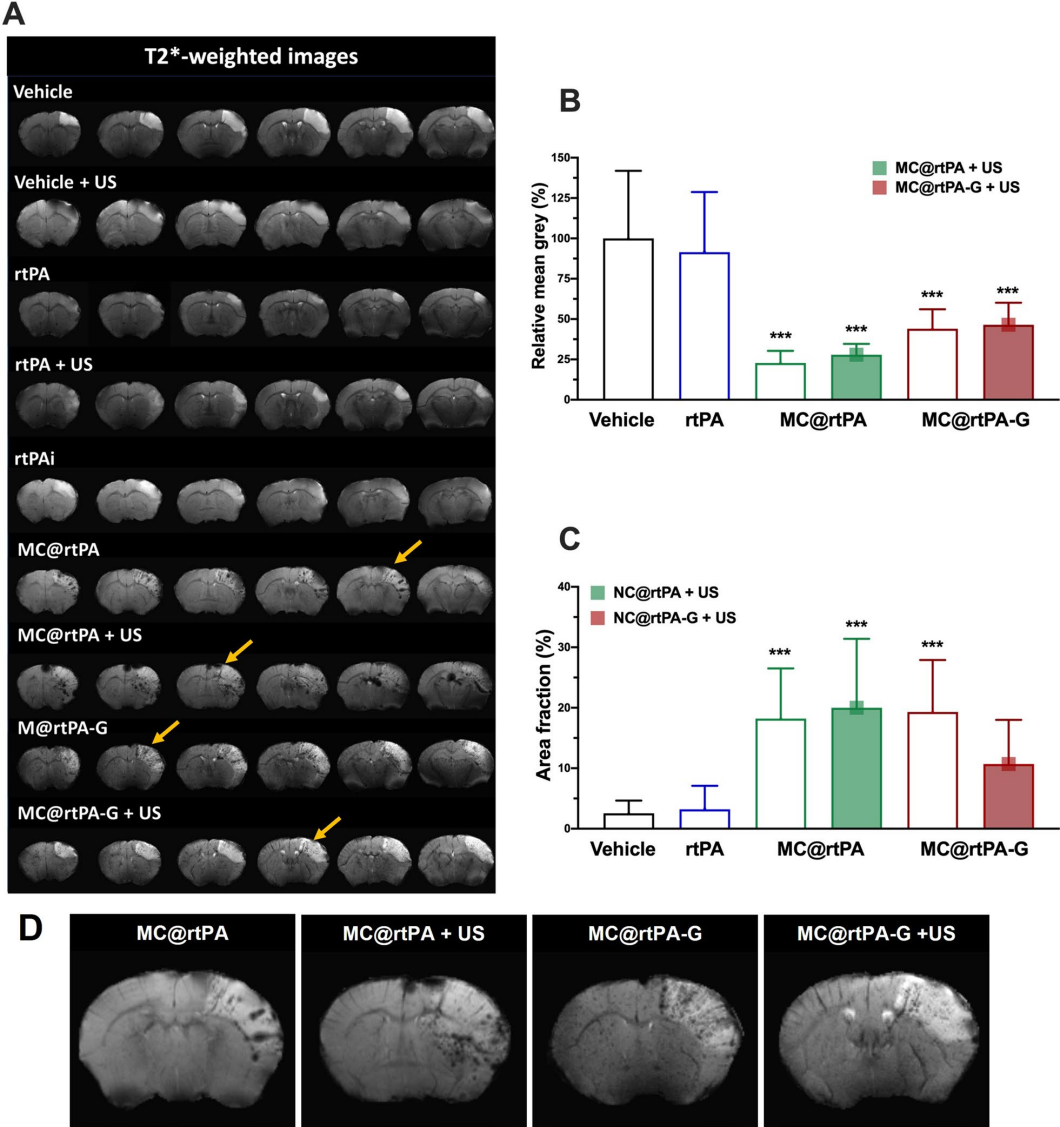
**A** Distribution of microcapsules in the brain analyzed by T2*-weighted images in the different experimental groups at 24 h after the treatment administration. SCs can be identified as hypo-signals (indicated with yellow arrows), mainly, in then ischemic region. **B** Quantification of the SCs accumulation determined grey value relative to the vehicle group. In each animal three independent measurements were performed. **C** Quantification of the SCs accumulation by area fraction. **D** MRI representation of an animal treated with saline (control) and the groups treated with the SCs, in which the hypo-signals could be observed as black spots. All data are represented as mean ± SD (n = 5 per group of treatment and three measurements per animal). In all data statistical analysis was assessed by the one-way ANOVA followed by post-hoc Brown-Forsythe test ***(P < 0.001)

Finally, based on magnetic labeling with ioNPs, the in vivo fate and biodistribution of SCs in the brain were investigated using MRI. Hyposignals in T2*-weighted images were quantified using two methods: mean gray value and area fraction. Quantification was performed on the ipsilateral hemisphere at 24 h (Fig. 6) and 3 days (Additional file 1: Fig. S7) after treatment administration.

Figure 6A shows the T2*-weighted images in which hypo-signals in the ischemic region are only observed in the groups treated with SCs. Two considerations lead us to believe that these signals correspond to the accumulation of SCs in the ischemic region and not to hemorrhagic lesions (as detected with the same MRI T2* sequence). The first is related to the echo time in T2*-weighted images, which is similar to previous studies in which mesenchymal cells labeled with magnetite NPs were intra-arterially injected into the brain [26]. Second, hemorrhagic lesions induce hypersignals that increase after several days due to the high amount of iron [27, 28]; however, in this case, the hypersignals decreased after 3 days of follow-up (Additional file 1: Fig. S7). Similar results were observed using the grey value MRI quantitative method (Fig. 6B). The highlighted area in the ischemic region was significantly increased in SCs treated groups, although significance was not achieved in the SC@rtPA-G group treated with US (Fig. 6C).

**Fig. 7:**
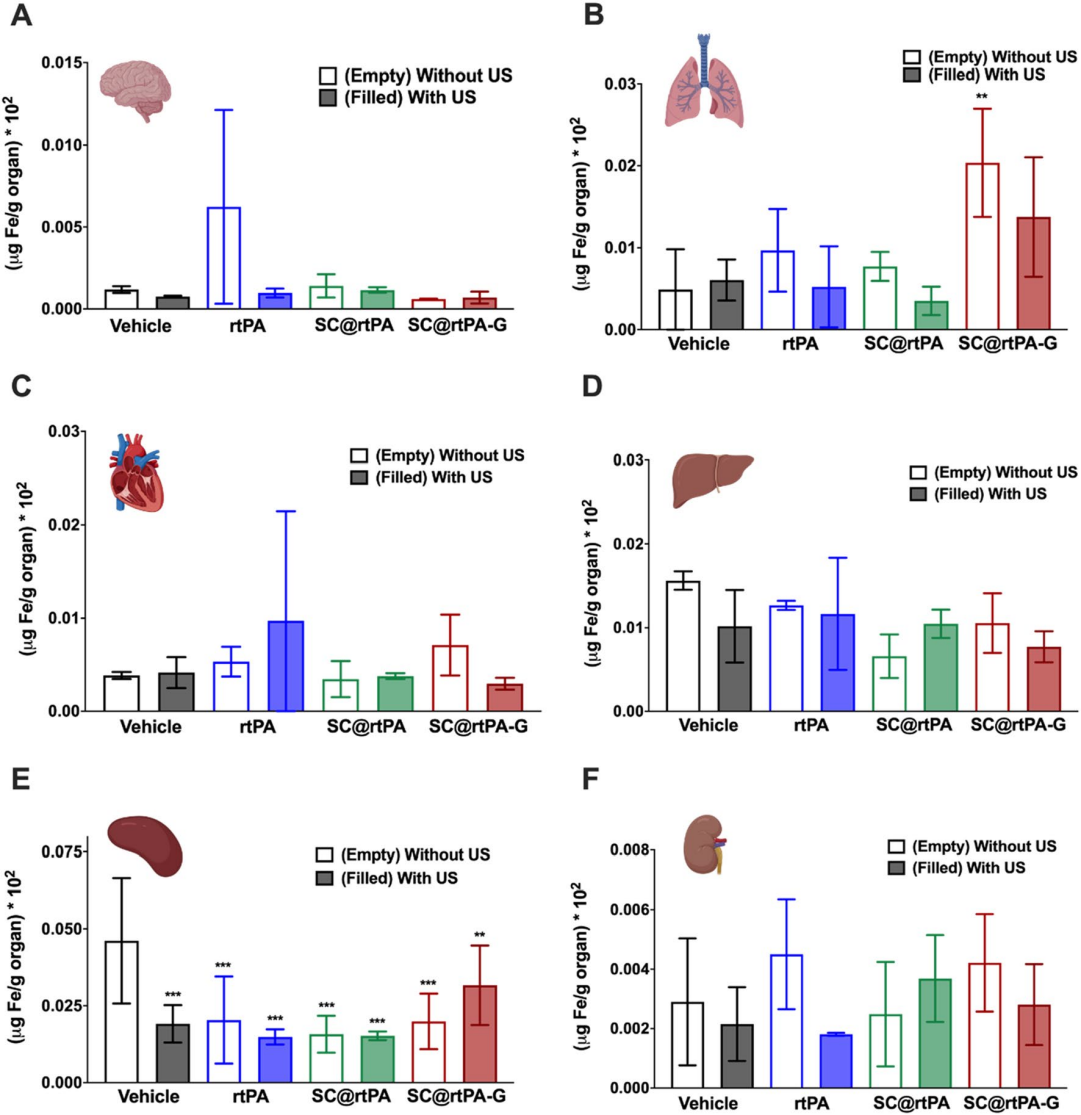
Biodistribution analysis of the SCs by inductively coupled plasma mass spectrometry (ICP-MS). Iron concentration of each treatment group in **A** brain, **B** lungs, **C** heart, **D** liver, **E** spleen and **F)** kidneys. Columns not filled in color represent the groups treated without US, while color-filled columns represent the groups treated with US. All data are represented as mean ± SD (n = 5 per group of treatment). In all data statistical analysis was assessed by a one-way ANOVA followed by post-hoc Brown-Forsythe test compared with the vehicle group (**P < 0.01); (***P < 0.001)

Complementary analysis of SCs distribution was performed in different organs (brain, lungs, heart, spleen, liver, and kidneys) by analyzing the iron content present as a result of SCs incorporation into the ioNPs. The data included in Fig. 7 show that the iron concentrations in different organs were very similar, independent of the treatment used.

## Discussion

We recently reported the synthesis and characterization of a new thrombolytic carrier based on polymeric LbL submicroparticles coated with gelatine. These SCs showed the capacity to maintain rtPA by protection from its main blood inhibitor and to release rtPA by US-triggering. Furthermore, analysis of renal and hepatic markers and MRI studies have demonstrated that these SCs do not induce toxicity or brain damage in healthy animals [19]. The present study, which complements these earlier findings, aimed to evaluate the biocompatibility and the therapeutic effect of our rtPA carriers under in vivo ischemic conditions. We first evaluated the rtPA US-triggered release in SCs with and without gelatine coating (SC@rtPA-G and SC@rtPA, respectively) and latter, their therapeutic effect.

In the first approach, we observed that only the SC@rtPA-G 1 mg/kg group responded to US exposure, allowing extension of the half-life of rtPA in blood compared with the group treated with the same dose of 1 mg/kg rtPA-free solution administered as a bolus. Meanwhile, the group treated with SC@rtPA without gelatine showed continuous rtPA release immediately after administration, independent of US application. Although gelatine was incorporated to the SCs to target the clot regions, these findings demonstrated the critical role of this protein in stabilizing encapsulation and maintaining the rtPA release response to US. This pharmacokinetic profile could be due to the presence of the arginine-glycine-aspartic acid sequence, which allows SCs to have low antigenicity, prolong their circulatory time, and evade the reticuloendothelial system [29, 30]. Encapsulation of rtPA in US-responsive gelatine complexes has also been reported as an alternative nano-delivery system for thrombolytic therapy; however, this design has been evaluated in myocardial infarction [31–33], but not in stroke pathology.

Interesting results were observed for the four groups treated with SCs. Independent of the rtPA release response, none were able to induce a therapeutic effect. Thus, the group treated with SC@rtPA-G + US presented a similar infarct as the ischemic control group, while the ischemic lesion was even higher in the other three groups. T2*-weighted images, used to detect the distribution of SCs in the brain, revealed the accumulation and aggregation of SCs, mainly in the ischemic regions. This particle aggregation indicates that SCs administration might produce secondary embolisms in the arteries, abolishing the thrombolytic effect of rtPA and increasing the ischemic lesion.

The evolution and distribution of T2*-weighted signals up to 3 days after ischemia confirm that the hypo-signals observed in the brain (black spots) are caused by the accumulation of SCs and eliminate the possibility of petechial haemorrhages [27, 28]. Distribution analysis of SCs in different organs (brain, lungs, heart, spleen, liver, and kidneys) showed no significant differences, so we cannot demonstrate that SC accumulation occurs only in the brain. In this analysis, it is important to note that ~ 128 µg Fe was administered per animal (5.1 mg/kg), a much lower dose than that used in other investigations, which was 700 µg Fe per animal (35 mg/kg) [34, 35], and therefore, this low (amount) resolution could be a limitation to detect differences among groups.

Different explanations could explain this particle accumulation in the ischemic region: (1) the number of capsules administered (2 to 5 × 10^9^) to reach the desired rtPA dose (1 mg/kg rtPA); (2) the properties of the SCs, such as their size (~ 600 nm) combined with poor elasticity, can induce reduced mobility through capillaries that contribute to aggregation [36, 37]; and (3) vessel tortuosity and ischemic processes, such as BBB disruption, local inflammatory response, or the reduction of the microvasculature lumen, which contributes significantly to particle aggregation [38]. This last observation is supported by the fact that we did not observe SCs accumulation in the brain using the same system and the same concentration in healthy animals.

The aggregation response could be due to the gelatine coating or US application on the ischemic region. However, this effect was independent of the gelatine coating, as the accumulation of SCs and increase in infarct volume were observed in both the SC@rtPA and SC@rtPA-G groups. Other studies that used gelatine complexes for rtPA encapsulation did not report aggregation in a heart ischemic model [32], which seems to confirm that this agent is safe for in vivo drug delivery. Regarding the use of US, the selected US parameters (0.72 W/cm^2^, 2 MHz) have been confirmed safe in rodent models [39] and humans [40]. In contrast, other clinical analyses have reported that US at diagnostic frequencies, as used in this study, could increase ischemic damage and the risk of cerebral haemorrhage in patients concomitantly treated with intravenous rtPA [41, 42]. In our study, the accumulation of SCs was not associated with the use of US and our safe analyses on BBB disruption confirm that the selected US parameters are biocompatible for in vivo drug triggering.

In line with these findings, risk of cerebral lesions after therapeutic interventions has been reported in stroke animal models after intra-arterial administration of mesenchymal stem [26] which demonstrates the vulnerability of the ischemic region against cell or nanoparticle-based treatments.

While the benefits of nanotechnology for stroke pathology have been widely reported, adverse effects are limited to the study of the toxicity of materials, inflammation response, or risk of tumoral formation [43]. Other studies have evaluated nanoparticle accumulation in systemic organs such as the liver, kidney, lung, or spleen, but only related to the analysis of particle clearance and drug bioavailability [44]. The use of submicroparticles for drug delivery has never been tested in the brain, and the analysis of other nanothrombolytic strategies has not reported side effects after in vivo administration for ischemic pathology. Therefore, to the best of our knowledge, this is the first study to report the risk-aggregation effect of a drug carrier in a translational stroke model.

## Conclusions

We evaluated the in vivo biocompatibility and therapeutic effect of thrombolytic sub-microparticles in a thromboembolic stroke model that reproduces the recanalization of rtPA treatment, as observed in humans. Despite promising results in healthy animals, at least under the dose and size conditions used in this study, the administration of these SCs is a risky therapeutic tool for stroke. Adverse effects related to the use of nanoparticles in vivo should be thoroughly studied to anticipate the limitations and orientate the design of new nanoparticles for translation to humans [45–47]. Future directions on the use of biomimetic nanoparticles, for instance, those with lower size and more elasticity, could be more appropriate as a therapeutic approach against stroke.

## Materials and methods

### Synthesis and characterization of the SCs

Synthesis and characterization of SCs were performed as described in our previous study [19]. Briefly, rtPA-loaded vaterite CaCO_3_ cores were synthesized by mixing CaCl_2_, Na_2_CO_3_, and rtPA (70 kDa, Alteplase, Actilyse™, Boehringer Ingelheim) labelled with fluorescein-5-isotiocyanate (FITC, Merck, Germany) for subsequent fluorescence quantification. LbL assembly was then carried out using poly (sodium 4-styrenesulfonate) (PSS, MW = 70 kDa, Merck) as a negatively charged polymer and poly(diallyldimethylammonium chloride) (PDADMAC, MW = 250–300 kDa, Merck) as a positively charged polymer. Iron oxide nanoparticles (ioNPs) were added to make these SCs suitable for MRI and ICP-MS analysis. Gelatine (from bovine skin, Merck) was incorporated as the final layer of SCs for VWF targeting. Finally, the CaCO_3_ cores were dissolved and eliminated by chelator treatment under mildly acidic conditions (EDTA, pH = 5.5) (Additional file 1: Scheme S1). The SC@rtPA and SC@rtPA-G batches were characterized before administration to validate the consistency and determine the number of particles required to administer the therapeutic dose of rtPA (1 mg/kg). Cargo (rtPA) loading was evaluated using fluorescence and flow cytometry to determine the concentration of SCs (SC/mL) using a Guava^®^ easyCyte BG HT flow cytometer (Millipore^®^, Spain). Additional information on the synthesis and characterization is provided in Additional file 1.

### Animal care

Male Swiss mice (Harlan Laboratories, Barcelona, Spain) weighing 25–30 g were used. Mice were kept in separate rooms under controlled temperature (22 ± 1 °C) and humidity (60 ± 5%) with a 12/12 h light/dark cycle for a week prior to surgery and up to 7 days after surgery. The animals had access to food and water ad libitum. All procedures were performed under anesthesia. Anesthesia was induced by inhalation of 5% sevoflurane in a nitrous oxide/oxygen mixture (70/30). The rectal temperature was monitored and maintained at 37 ± 0.5 °C using a feedback-controlled heating system. At the end of the procedure, the mice were sacrificed under deep anesthesia (8% sevoflurane). The experimental protocol was approved by the Animal Experimental Committee of the University of Santiago de Compostela, Spain. The animal experiments were conducted under the procedure number: 15010/2019/004 according to the Spanish and EU rules (86/609/CEE, 2003/65/CE, 2010/63/EU, RD 1201/2005 AND RD 53/2013).

### Ultrasound-triggered in vivo delivery of rtPA

To validate rtPA encapsulation in the SCs and US-induced drug release with and without gelatine (SCs@rtPA-G and SCs@rtPA, respectively), treatments were administered first to healthy animals (n = 3/group). All treatments were administrated as a volume of 0.2 mL through the femoral vein. Blood rtPA plasmatic activity was determined (Sensolyte AMC t-PA Activity Assay, Anaspec, France) in basal conditions (before treatment administration) and at 1, 5, 15 and 40 min after administration. Blood was sampled by inserting a cannula into the carotid artery. In these groups, external US radiation (2 MHz, 0.72 W/cm^2^) was continuously applied using a transcranial Doppler (Compumedics DWL, Germany) in the abdominal region for a period of 40 min after treatment administration.

### In vivo therapeutic effect of SCs in stroke models

The same experimental groups (with and without US) of healthy animals were later evaluated under ischemic conditions (n = 5/group). The thromboembolic stroke model was induced by injection into the MCA of mice, as originally described by Orset et al. [48]. The mice were placed in a stereotaxic frame, the skin between the right ear and eye was cut, the temporal muscle was retracted, and the temporal and parietal bones were exposed. A small craniotomy was performed over the artery bifurcation, the meninges were cut using a 25 G needle (BD Microlance, Italy), and the MCA was exposed. A micropipette (tip size: 20–40 µm) made using hematologic glass capillaries (World Precision Instruments, Inc. USA) using a puller (Sutter Instruments, USA), was pneumatically filled with 1.5 µL of 1.5 U/µL thrombin (Murine Thrombin 0.05 mg MIIA. Stago-BNL, Belgium). The micropipette was placed in a micromanipulator and 1 μL of thrombin solution was injected into the lumen of the artery bifurcation to induce clot formation. The micropipette was removed 15 min after the clot was stabilized.

CBF was monitored using a Periflux 5000 laser Doppler perfusion monitor (Perimed AB, Sweden) by placing the probe (Perimed, Sweden) in the parietal territory of the MCA. Basal CBF was measured for at least 5 min throughout the experiment. Artery occlusion was considered successful when the CBF decreased by more than 80% relative to the basal flow and reperfusion when at least 40% of the basal CBF was recovered. Thirty minutes after thrombin occlusion, different treatments were administered intravenously into the tail vein. For this intervention, a small incision was made in the tail of the animal, the skin was excised, and the tail vein was exposed. A 30 G needle (BD Microlance, Italy) was used for every treatment, and the puncture was rapidly closed to prevent bleeding. In the animals in which the treatment was administered as an infusion, the 30 G needle was placed in a catheter (polythene tube, 0.28 mm internal diameter, 0.61 mm outside diameter; Smiths, Spain) and the injection rate was controlled using a micro-pump (4.5 µL/min). In the groups treated in combination with US, the setup was similar to the one used in healthy animals (2 MHz, 0.72 W/cm^2^ during 40 min after stroke induction), but US were applied on the ischemic side of the animal head.

### Ultrasound safety analysis in healthy and ischemic animals

The selected US parameters (0.72 W/cm^2^, 2 MHz) have been confirmed to be safe in rodent models [39] and are within the range of parameters used in clinical transcranial Doppler sonography [40]. To guarantee the safety of the US parameters (0.72 W/cm^2^, 2 MHz) in our animal model, we analyzed BBB disruption after US application using the EB technique under healthy and ischemic conditions (n = 5 per group). This assay is based on the ability of EB to bind serum albumin. Extravasation of albumin to the cerebral parenchyma is correlated with BBB disruption [49]. To perform this study, EB was injected at 4 mg/kg in four experimental groups: healthy and ischemic animals, with and without US.

In healthy animals, EB was injected prior to US application. In the ischemic group, EB was injected 30 min after thrombin injection and immediately after US application. In all the experimental groups, US was applied for 40 min. The mice were euthanized 24 h after EB injection. The brains were removed, divided into ipsilateral and contralateral hemispheres, and weighed. The hemispheres were frozen immediately in liquid nitrogen and stored at − 80 °C. For analysis, brain samples were placed in a stove at 55 °C in 1 mL of *N*-methylformamide for 48 h. The samples were then centrifuged (9000 rpm, 20 min), and the absorbance due to the EB present in the supernatant was measured with a plate reader (630 nm). The extravasated dye content was normalized to the brain weight (µg/g).

### Magnetic resonance imaging analysis

MRI was performed at 24 h and 3 days after ischemic induction to evaluate the infarct size and fate of SCs.

MRI studies were conducted using a 9.4 T horizontal bore magnet (Bruker BioSpin, Ettligen, Germany) with 12 cm wide actively shielded gradient coils (440 mT/m). Radiofrequency transmission was achieved with a birdcage volume resonator, and the signal was detected using a two-element arrayed surface coil (RAPID Biomedical, Germany), positioned over the head of the animal, which was fixed with a tooth bar, earplugs, and adhesive tape. The respiratory frequency and body temperature were monitored during the experiments. The transmission and reception coils are actively decoupled from each other. Gradient-echo pilot scans were performed at the beginning of each imaging session to accurately position the animals inside the magnet bore.

The progression of ischemic lesions and infarct volumes were determined from T2-maps calculated from the T2-weighted images. Ischemic lesions were determined by counting pixels with apparent T2-map values above the threshold in the ipsilateral brain hemisphere. In healthy mice, the T2-map values of the brain are over 50 ms. In the ipsilateral ischemic hemisphere, hyperintensity on the T2-map determined the analysis of ischemic damage (T2 map values > 60 ms) [50]. T2-weighted images were acquired using a multi-slice multi-echo (MSME) sequence with an 11 ms echo time (TE), 2.8 s repetition time (TR), 12 echoes with 11 ms echo spacing, FA of 180º, 2 averages, 50 kHz spectral bandwidth (SW), 16 slices of 0.5 mm, 19.2 × 19.2 mm^2^ field of view (FOV) with saturation bands to suppress the signal outside this FOV, a matrix size of 256 × 256 (isotropic in-plane resolution of 75 μm/pixel × 75 μm/pixel), and implemented without fat suppression. The acquisition time was 23 min.

The distribution of SCs in the brain was evaluated by T2*-weighted images. T2*-weighted images were acquired using a multi-gradient-echo sequence (MGE) with a 5 ms TE, 1.2 s TR, 8 echoes with 4.5 ms echo spacing, 100 kHz spectral bandwidth, FA of 20°, 16 slices of 0.55 mm, 2 averages, 19.2 × 19.2 mm^2^ FOV with saturation bands to suppress signal outside this FOV, and a matrix size of 256 × 256 (isotropic in-plane resolution of 75 μm/pixel × 75 μm/pixel) and implemented with the fat suppression option. The acquisition time was 10 min.

The hypo-signals in the T2*-weighted images were quantified using two different methods: mean grey value and area fraction [51]. ImageJ software (Rasband WS, National Institutes of Health, Bethesda, MD, USA) was used to quantify these signals. In both methods, we performed quantification in all the groups treated with SCs, in which the hypo-signals appeared, and as a control, we performed the quantification in all the animals treated with the vehicle and rtPA. Quantification was performed on three different slides for each animal. Additional details regarding quantification are provided in Additional file 1: Fig. S8.

### Analysis of SCs accumulation in organs

Biodistribution of SCs in different organs (brain, lungs, heart, spleen, liver, and kidneys) was determined by analyzing the iron concentration using ICP-MS, as described elsewhere [19]. Briefly, the samples were weighed and completely digested by the addition of 5 mL of ultra-pure (67 w/v %) HNO_3_ (Fisher Chemical, Massachusetts, USA) under constant agitation in 50 mL Falcon tubes. Digestion took place over 48 h at 21 °C until no organic residues remained. This liquid solution was then further digested using aqua regia consisting of three parts concentrated ultra-pure (35 wt %) HCl (Fisher Chemical) and one part of ultra-pure (67 wt %) HNO_3_. For this final digestion, 100 µL of a well-mixed sample solution was added to 300 µL of aqua regia and agitated for at least 2 h (i.e., the sample was diluted by a factor of 4). The samples had to be diluted tenfold using a low matrix of 2% (w/v) HCL, which was adapted to the material, to use on the ICP-MS instrument.

### Statistical analysis

All data are presented as the mean and SD of the mean (mean ± SD). The data were first examined to assess the distribution using D’Agostino and Pearson omnibus normality tests. A *t*-test or one-way analysis of variance (ANOVA) followed by post-hoc Brown-Forsythe evaluation was used to detect significant differences between groups. Statistical significance was set at P < 0.05. Statistical analysis and graphical representation were performed using GraphPad Prism 8.0.

## Supplementary Information

**Additional file 1: **Scheme 1. Schematic representation of the layer-by-layer process. **Figure S1**: SEM micrographs of the samples after drop casting on Si substrates. **Figure S2**: TEM micrographs of the samples after drop casting on top of a copper grid coated with a layer of carbon**. Figure S3**: DLS graphs **A**) intensity, **B**) number and **C**) ζ-potential of core@rtPA (gray), SC@rtPA (blue) and SC@rtPA-G (pink) measured in Milli Q water. **Table S1:** Mean average hydrodynamic diameters and ζ-potential values. **Figure S4**: rtPA-FITC calibration curve. **Figure S5**: SCs dispersion analysis by flow cytometry. **Figure S6** **A**) Ischemic lesion (white brain region) determined by T2-weighted at 3 days after the treatment administration in the 9 experimental groups: vehicle, vehicle with ultrasound (US), rtPA (1 mg/kg as bolus), rtPA with US (1 mg/kg as bolus), rtPAi (rtPA 10 mg/kg as bolus and infusion), SC@rtPA (1 mg/kg as bolus), SC@rtPA with US (1 mg/kg as bolus), SC@rtPA-G (1 mg/kg as bolus) and SC@rtPA-G with US (1 mg/kg as bolus). **B**) Analysis of the infarct volume at 24 hours after treatment administration. **Figure S7** **A** Distribution of the microcapsules in brain analyzed by T2*-weighted in the different experimental groups at 3 days after the treatment administration. SCs can be identified as hypo-signals (indicated with yellow arrows), mainly, in then ischemic region. **B** Quantification of the SCs accumulation determined by grey value relative to the vehicle group. In each animal three independent measurements were performed. **C** Quantification of the SCs accumulation by area fraction. **D** MRI representation of a animals treated with saline (control) and the groups treated with the SCs, in which the hypo-signals could be observed as black spots  **Figure S8**. Methods to quantify the hypo-signals in T2*-weighted images.

## Data Availability

Data sharing is applicable to this article.
