# Supplementary material for: Sonosensitive capsules for brain thrombolysis increase ischemic damage in a stroke model
Source: J Nanobiotechnology. 2022 Jan 21;20:46. doi: 10.1186/s12951-022-01252-9 (PMC8780814; doi:10.1186/s12951-022-01252-9)
Supplement: Supplementary file 1 — Additional file 1: Scheme 1. Schematic representation of the layer-by-layer process. Figure S1: SEM micrographs of the samples after drop casting on Si substrates. Figure S2: TEM micrographs of the samples after drop casting on top of a copper grid coated with a layer of carbon. Figure S3: DLS graphs A) intensity, B) number and C) ζ-potential of core@rtPA (gray), SC@rtPA (blue) and SC@rtPA-G (pink) measured in Milli Q water. Table S1: Mean average hydrodynamic diameters and ζ-potential values. Figure S4: rtPA-FITC calibration curve. Figure S5: SCs dispersion analysis by flow cytometry. Figure S6 A) Ischemic lesion (white brain region) determined by T2-weighted at 3 days after the treatment administration in the 9 experimental groups: vehicle, vehicle with ultrasound (US), rtPA (1 mg/kg as bolus), rtPA with US (1 mg/kg as bolus), rtPAi (rtPA 10 mg/kg as bolus and infusion), SC@rtPA (1 mg/kg as bolus), SC@rtPA with US (1 mg/kg as bolus), SC@rtPA-G (1 mg/kg as bolus) and SC@rtPA-G with US (1 mg/kg as bolus). B) Analysis of the infarct volume at 24 hours after treatment administration. Figure S7 A Distribution of the microcapsules in brain analyzed by T2*-weighted in the different experimental groups at 3 days after the treatment administration. SCs can be identified as hypo-signals (indicated with yellow arrows), mainly, in then ischemic region. B Quantification of the SCs accumulation determined by grey value relative to the vehicle group. In each animal three independent measurements were performed. C Quantification of the SCs accumulation by area fraction. D MRI representation of a animals treated with saline (control) and the groups treated with the SCs, in which the hypo-signals could be observed as black spots Figure S8. Methods to quantify the hypo-signals in T2*-weighted images. [file 12951_2022_1252_MOESM1_ESM.docx]

**SUPPLEMENTARY INFORMATION**

**Synthesis of the SC**

In this work the synthesis had to be scaled up in order to reach the desired dose of rtPA.

**rtPA labelling**: rtPA was labelled with fluorescein-5-isothiocyanate (FITC, #1245460250) at pH 8 in phosphate-buffered saline (PBS) and left to react protected from light for 4 h; ca. 25 dyes per protein were used. Then a size exclusion column (PD-10, MWCO = 5000 Da; #GE17-0851-01) was used to purify the labeled protein from the excess of free dye and in case of rtPA, partly, from the excipients (i.e., arginine and polysorbate 80). The dye-protein was collected according to the manufacturer’s instructions. To avoid the precipitation of rtPA-FITC, the column was equilibrated with 3.5 mg/mL arginine in PBS (10 mM, pH 7.4). The same buffer was used to collect the rtPA-FITC. Without this concentration of arginine, rtPA precipitation inside the PD-10 column occurred quickly, as could be observed simply by visual inspection. The final rtPA-FITC concentration was determined by using the Bradford assay (Pierce TM Coomassie Plus Assay Kit; ThermoFisher #23236). Then, the sample of dye-labeled protein with a known concentration was used to perform a calibration curve of protein concentration *versus* fluorescence signal (exc./em. 485 /535 nm). This calibration curve was used to determine the final rtPA-FITC loading onto the SCs. The fluorescence was measured using a plate reader TECAN Infinite 2000.

**Cores synthesis**: cores were synthesized as follows: 1 mL of 20 mM CaCl_2_·2H₂O (#223506, Sigma-Aldrich) solution with PVSA (0.1 mM, #278424, Sigma-Aldrich) was mixed under continuous stirring (550 rpm) in a beaker with a magnetic stirrer. Then, 20 μL of rtPA-labeled (1 mg/mL) was added. After 5 minutes stirring at room temperature, 1 mL of 20 mM Na_2_CO_3_ (#S7795, Sigma-Aldrich) solution was added rapidly, the beaker was covered, and the solution was mixed (550 rpm) for 30 minutes at room temperature in a magnetic stirrer.

Ten syntheses of cores were mixed and obtained by precipitation at 3x10^3^ rcf (10 minutes). After centrifugation, the precipitate was washed with a sodium bicarbonate buffer solution (0.1 M NaHCO_3_, pH 9) and precipitated one more time at 3×10^3^ rcf (10 minutes).

Finally, the cores were resuspended in 10 mL of sodium bicarbonate buffer solution and immediately used for layer by layer (LbL) deposition of polyelectrolytes, iron oxide nanoparticles (ioNPs) and gelatine.

**Layer-by-layer**: for LbL, solutions of PSS (20 mg/mL with 0.5 M NaCl, #243051, Sigma-Aldrich), PDADMAC (20 mg/mL with 0.5M NaCl, #409022, Sigma-Aldrich), ioNPs (0.5 mg/mL in milliQ water, 0.5 M NaCl), and gelatine (7 mg/mL) were used. Briefly, 20 mL of cores were added to the PSS solution (20 mL). Then, the solution was mixed in a shaker for 10 minutes, and the cores precipitated at 3x10^3^ rcf (10 minutes) and washed/precipitated one more time with buffer. Then, the PDADMAC solution was used (same conditions as the PSS deposition) to form the next layer, and so forth. In addition, a solution of ioNPs (0.5 mg/mL in milliQ water, 0.5 M NaCl) was used instead of the PSS solution, to form a layer of NPs after the first PSS/PDADMAC bilayer. Then, another bilayer of PDADMAC/PSS was performed.

Alternatively, an outermost layer of basic gelatine (7 mg/mL) was formed. 0.6 g of basic gelatine (#G9391, Sigma-Aldrich) were dissolved in 12 mL of milliQ water at 50 °C under stirring. When the gelatine was completely dissolved, 12 mL of acetone were rapidly added to the solution. After 10 s, part of the gelatine precipitated in the form of a dense solid. This purified gelatine was dried, and a solution of 7 mg/mL in water was prepared and used for the LbL. The CaCO_3_ cores coated with the polyelectrolytes, ioNPs and gelatine were exposed to ethylenediaminetetraacetic acid (EDTA, 0.02 M, pH 5.5) for 1 hour to produce the hollow sub-micrometric sonosensitive capsules (SCs) loaded with rtPA. Finally, the SCs were washed/precipitated twice at 5×10^3^ rcf (5 minutes), see **Scheme 1**.


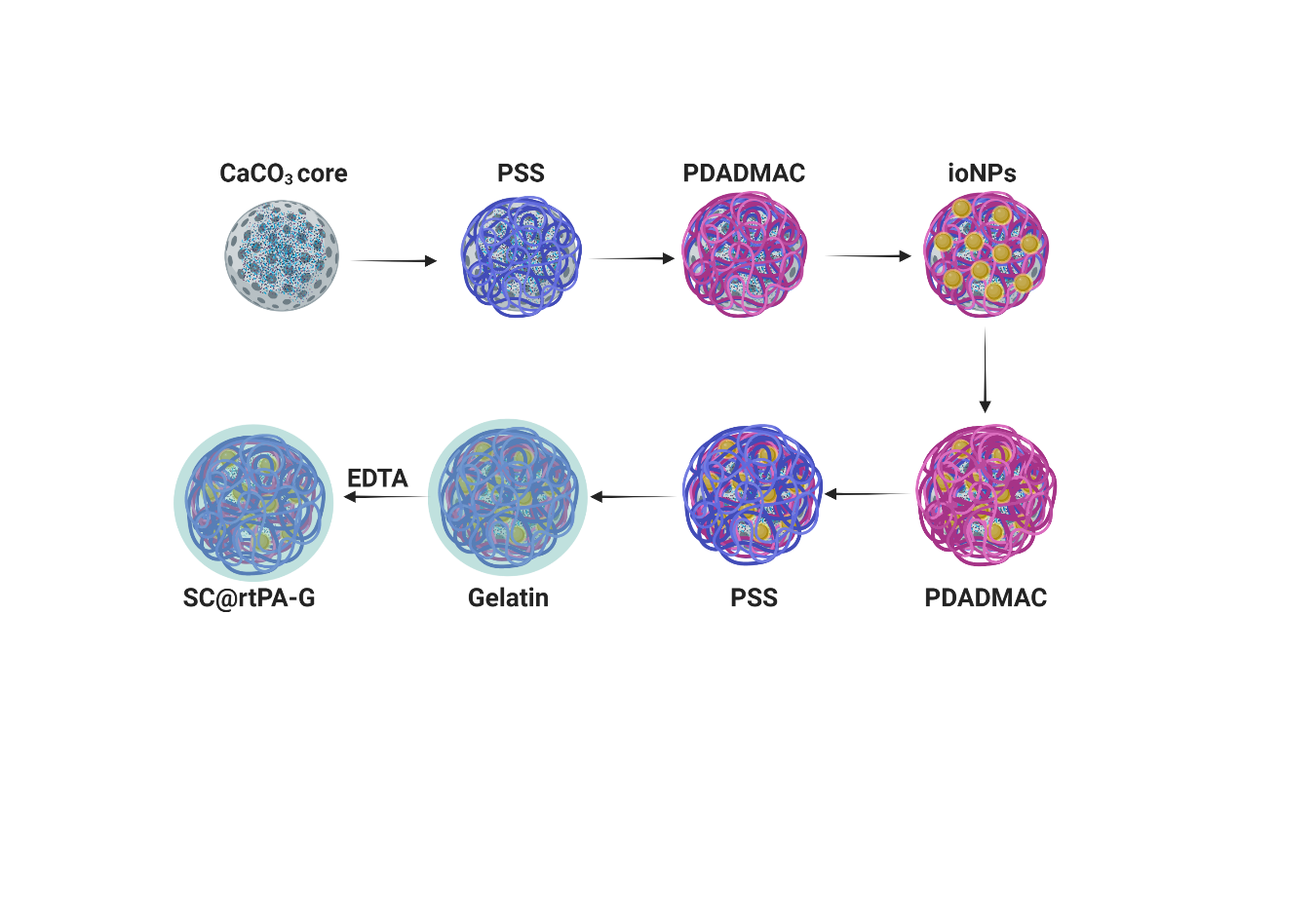


**Scheme 1.** Schematic representation of the layer-by-layer process. PSS (poly (sodium 4-styrenesulfonate); PDADMAC (poly(diallyldimethylammonium chloride); ioNPs (iron oxide nanoparticles); EDTA (ethylenediamine tetraacetic acid); SC@rtPA-G (nanocapsules encapsulating rtPA with gelatine).

**Characterization of rtPA loaded SCs**

**Scanning Electron Microscopy (SEM):** The morphology and mean diameter of macromolecule-loaded (rtPA) cores was analyzed by SEM. Selected examples are shown in **Fig. 1** and **Fig. S1**. After LbL, the morphology of SCs loaded with rtPA was also analyzed by SEM after drop casting on Si substrates. Characterization parameters were similar as previously reported [16].


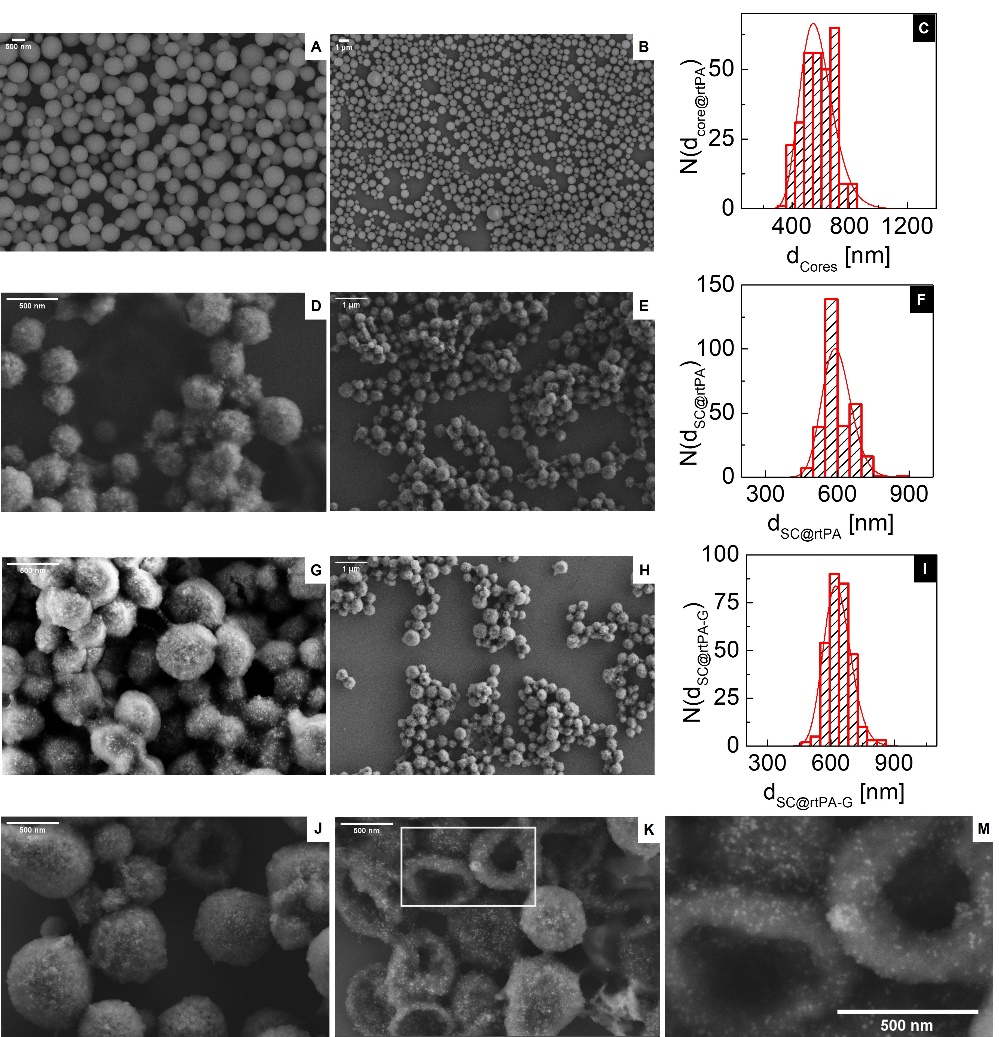


**Figure S1**: SEM micrographs of the samples after drop casting on Si substrates. **A** and **B**) High and low magnification SEM micrographs of cores (scale bars are 500 nm and 1 μm, respectively). **C**) Size histogram (d_core@rtPA_: mean diameter of core@rtPA, rtPA loaded vaterite CaCO_3_ cores) obtained after measuring the diameter of 300 core@rtPA using ImageJ; _dcore@rtPA_ = 580 ± 111 nm. **D** and **E**) High and low magnification SEM micrographs of SC@rtPA (no gelatine) before core removal (scale bars are 500 nm and 1 μm, respectively). **F**) Size histogram (d_SC@rtPA_: mean diameter of SC@rtPA) obtained after measuring the diameter of over 300 SC@rtPA using ImageJ; d_SC@rtPA_ = 599 ± 60 nm. **G** and **H**) High and low magnification SEM micrographs of SC@rtPA-G (gelatine) before core removal (scale bars are 500 nm and 1 μm, respectively). **I**) Size histogram (d_SC@rtPA-G_: mean diameter of SC@rtPA-G) obtained after measuring the diameter of over 300 SC@rtPA-G using ImageJ; d_SC@rtPA-G_ = 632 ± 65 nm. **J, K** and **M)** (magnified image of the region indicated in **K**) SEM micrographs of SC@rtPA-G after core removal; ioNPs are distinguishable as bright dots in the polymer shell of collapsed the SCs.

**Transmission Electron Microscopy (TEM):** The morphology and mean diameter of macromolecule-loaded (rtPA) cores was analyzed by TEM. TEM images were acquired with a JEOL JEM-1011 microscope by deposition of the sample on top of a copper grid coated with a layer of carbon. Selected examples are shown in **Fig. 1** and **Fig. S2**. After LbL, the morphology of SCs loaded with rtPA was also analyzed by TEM after drop casting on top of a copper grid coated with a layer of carbon.


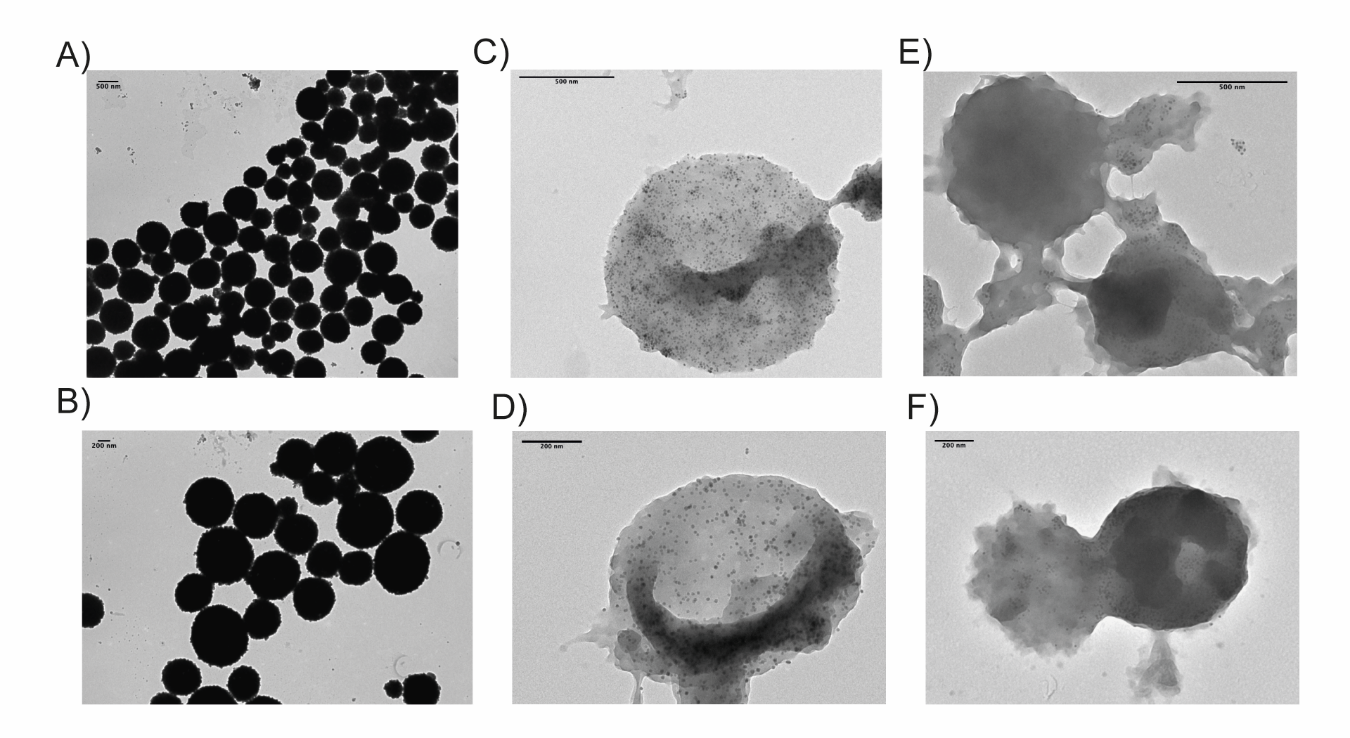


**Figure S2**: TEM micrographs of the samples after drop casting on top of a copper grid coated with a layer of carbon**. A, B)** TEM micrograph of cores (scale bars are 500 nm and 200nm respectively). **C, D)** TEM micrograph of SC@rtPA (no gelatine) after core removal (scale bars are 500 nm and 200nm respectively). **E, F**) TEM micrographs of SC@rtPA-G after core removal (scale bars are 500 nm and 200nm respectively); ioNPs are distinguishable as dark dots in the polymer shell of collapsed the SCs.

**Dynamic Light Scattering (DLS)**: We used a Malvern Zetasizer Nano ZSP to determine hydrodynamic sizes and ζ-potential values of cores and SCs. Measurements were performed in MilliQ water with a pH between 5.8 to 6. Graph representations of DLS and ζ-potential measurements are shown in **Fig. S3**. Mean average values (three independent measurements) of selected samples are provided in the following **Table S1**. In detail, we show values for the SCs system: core@rtPA, SC@rtPA and SC@rtPA-G after EDTA. In **Table S1**, orange-filled boxed are the results of the characterization of the samples from our previous report using the same SCs. Please note, that in this case the original cores were *ca.* 100 nm bigger, therefore the final size increases consistently, and the changes in the ζ-potential values are following the same trend. That variation in size we related to the fact that we scale-up the core-synthesis (we scale the written protocol up to 20 times in order to generate enough quantities for the *in vivo* studies).


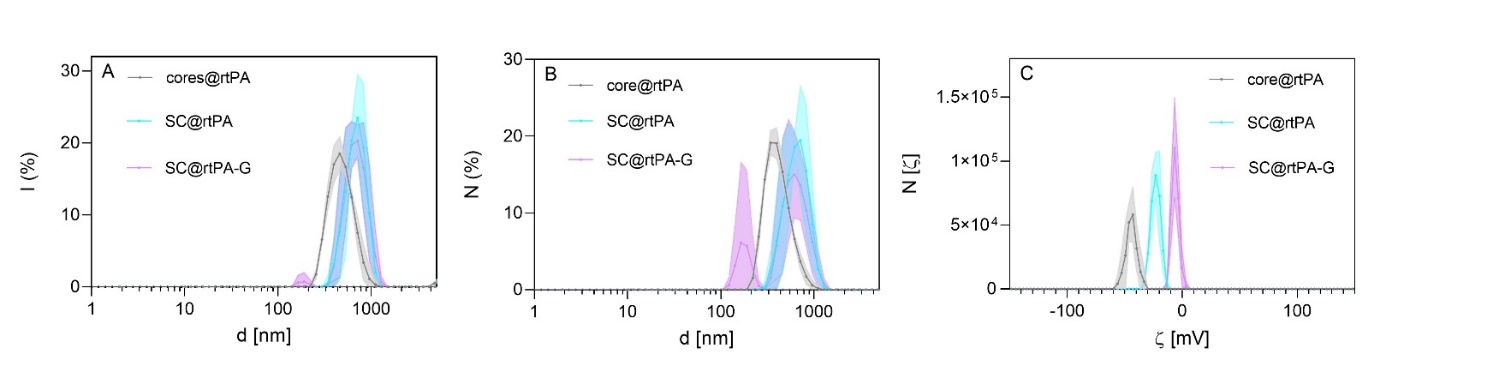
**Figure S3**: DLS graphs **A**) intensity, **B**) number and **C**) ζ-potential of core@rtPA (gray), SC@rtPA (blue) and SC@rtPA-G (pink) measured in Milli Q water.

| **Sample** | **d_H,I_ (nm)** | **d_H_ (nm)** | **PDI** | **ζ-potential (mV)** |
| --- | --- | --- | --- | --- |
| core@rtPA | 583.1 ± 50.4 | 421.5 ± 14.8 | 0.22 ± 0.04 | -44.4 ± 2.1 |
| SC@rtPA (before core removal) | 691.5 ± 74.2 | 659.9 ± 94.5 | 0.37 ± 0.03 | -22.7 ± 1.0 |
| SC@rtPA-G (after core removal) | 698.6 ± 79.0 | 562.7 ± 140.9 | 0.40 ± 0.06 | -6.1 ± 0.4 |
| core@rtPA | 648 ± 26 | 592 ± 17 | 0.09 | -48.9 ± 5.5 |
| SC@rtPA-G (before core removal) | 834.6 ± 36.3 | 799.6 ± 32.3 | 0.18 | -39.1 ± 5.1 |
| SC@rtPA-G (after core removal) | 853.7 ± 48.5 | 835.7 ± 43.9 | 0.23 | 15.6 ± 3.6 |

**Table S1:** Mean average hydrodynamic diameters and ζ-potential values. d_H,I_ and d_H_ refer to the mean average hydrodynamic diameter from the intensity and number DLS distributions. PDI refers to polydispersity index. Standard deviation values were calculated from three independent measurements.

**rTPA loading quantification in SCs:** Quantification of the encapsulated cargo, rtPA-FITC, was done in all purified SC@rtPA samples as an indirect measurement of rtPA concentration based on fluorescence, using the calibration curve (**Fig. S4**) prepared as previously described *in section rtPA labelling*.

**Figure S4**: rtPA-FITC calibration curve. Correlation between rtPA concentration (as determined by the Bradford assay) and fluorescence intensity. Linear fit was used to calculate unknown concentrations from fluorescence readings of SC@rtPA-FITC samples.

Then **flow cytometry (FC)** measurements were performed to determine the SCs concentration (*i.e.*, SC/mL) as described in *C. Correa-Paz, et al.* [19]. The scattering (forward and side) and fluorescence intensity (see **Fig. S5**) of the SCs samples were measured with a Guava® easyCyte BG HT flow cytometer (Millipore®), at a constant flow rate of 0.12 μL/s, using a blue laser emitting at 488 nm and a green laser emitting at 532 nm as excitation sources. The number of events measured in the corresponding SC gate divided by the acquired volume gives the final SC concentration. Finally, quantification of rtPA per SC was done in all cases as an indirect fluorescent measurement of rtPA mass and the quantification of the SC per mL obtained by Flow Cytometry (**Table 1**).


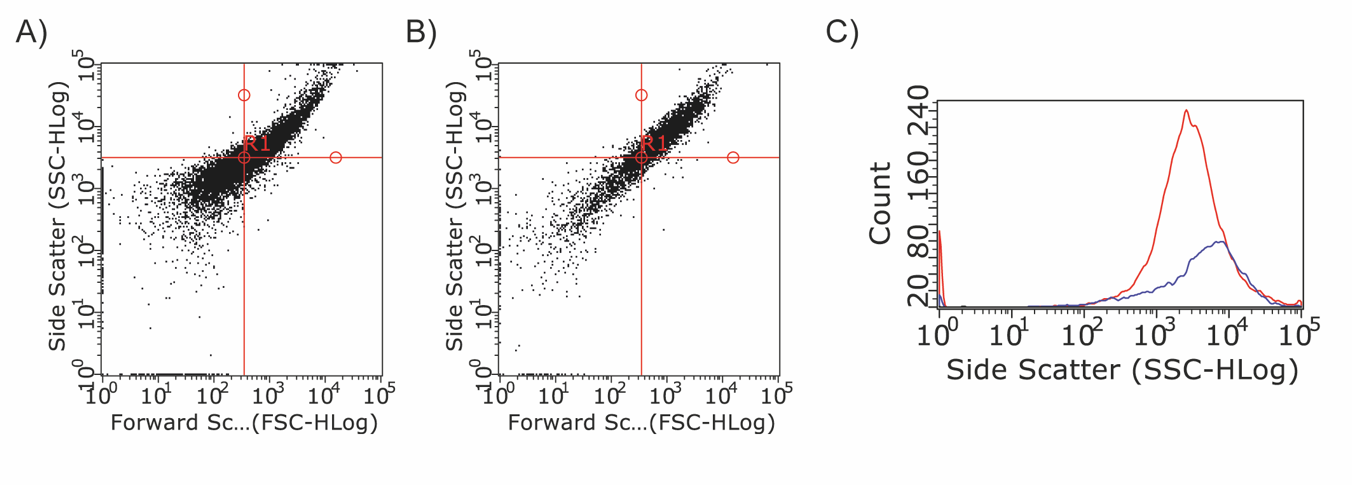


**Figure S5**: SCs dispersion analysis by flow cytometry. Scatter density plots of side scattering signal versus forward scattering signal for SCs (A) and SCs with gelatine (B). C) Representative histograms of side scattering distribution of SCs (red) and SCs with gelatine (blue)

**Infarct volumes 3 days after ischemia**


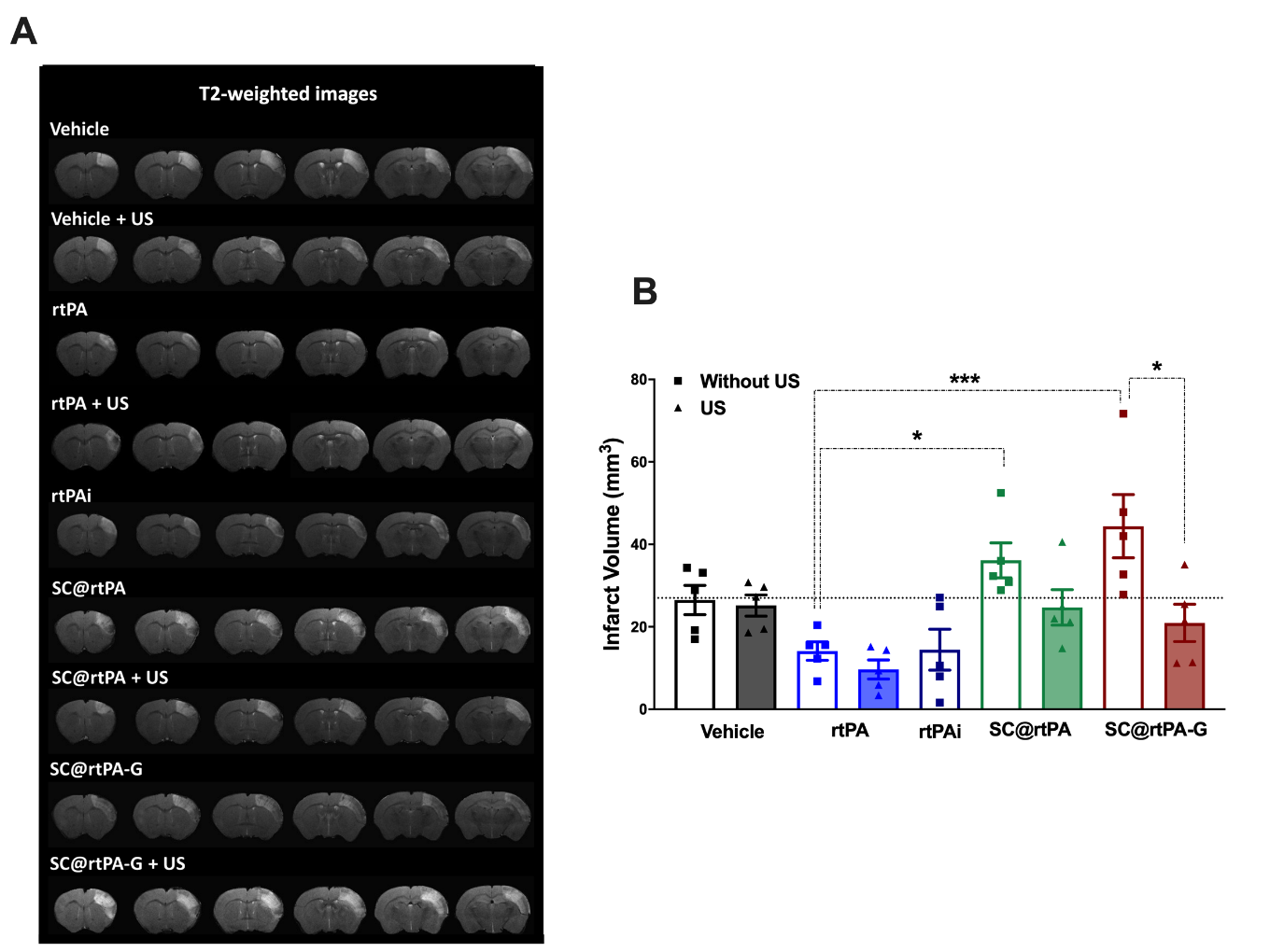


**Figure S6** **A**) Ischemic lesion (white brain region) determined by T2-weighted at 3 days after the treatment administration in the 9 experimental groups: vehicle, vehicle with ultrasound (US), rtPA (1 mg/kg as bolus), rtPA with US (1 mg/kg as bolus), rtPAi (rtPA 10 mg/kg as bolus and infusion), SC@rtPA (1 mg/kg as bolus), SC@rtPA with US (1 mg/kg as bolus), SC@rtPA-G (1 mg/kg as bolus) and SC@rtPA-G with US (1 mg/kg as bolus). **B**) Analysis of the infarct volume at 24 hours after treatment administration. The dotted lines represent the median of the vehicle group. The square symbols and empty columns represent the groups treated without ultrasound, while the triangles and colored columns are the groups treated with ultrasound. All data are represented as mean ± SD (n=5 per group of treatment). In all data statistical analysis was assessed by the one-way ANOVA followed by post-hoc Brown-Forsythe test. *(P<0.05), **(P<0.01); ***(P<0.001).

**T2*-weighted images at 3 days**


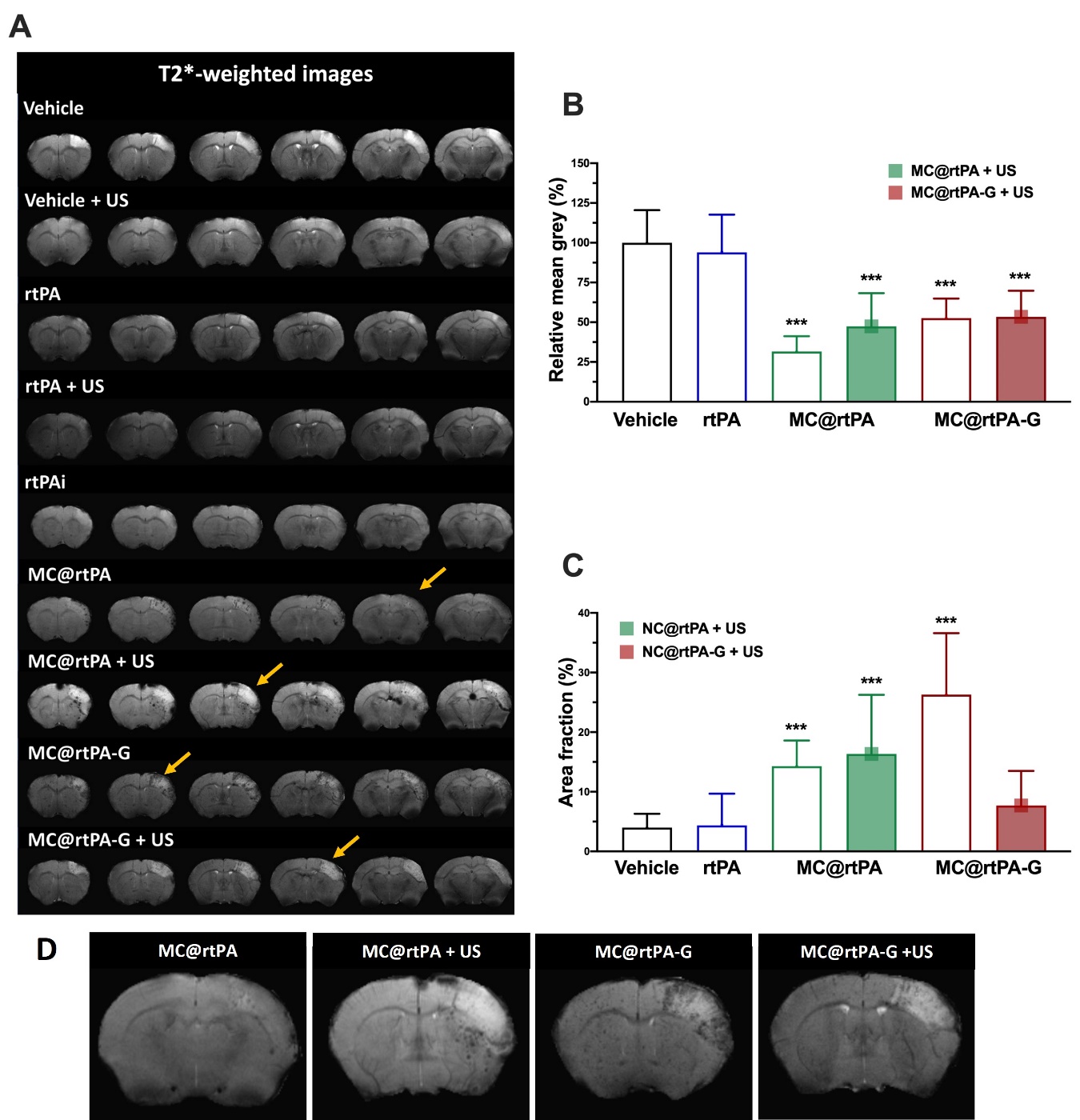


**Figure S7** **A**) Distribution of the microcapsules in brain analyzed by T2*-weighted in the different experimental groups at 3 days after the treatment administration. SCs can be identified as hypo-signals (indicated with yellow arrows), mainly, in then ischemic region. **B**) Quantification of the SCs accumulation determined by grey value relative to the vehicle group. In each animal three independent measurements were performed. **C**) Quantification of the SCs accumulation by area fraction. **D**) MRI representation of a animals treated with saline (control) and the groups treated with the SCs, in which the hypo-signals could be observed as black spots All data are represented as mean ± SD (n=5 per group of treatment and three measurements per animal). In all data statistical analysis was assessed by the one-way ANOVA followed by post-hoc Brown-Forsythe test ***(P<0.001).

**Quantification of T2*-weighted images**

The quantification of T2*-weighted images was performed in all the animals treated with SCs in which the hypo-signals appeared, and in all the animals treated with rtPA and the vehicle. The measurements were done in 3 different slides by two methods, grey value, and area fraction. In the first one, we selected a small hypo-signal area in the ischemic region using the T2* maps. Mean grey value is the average of the grey values of all the pixels in the selection divided by the number of pixels. The quantification was performed in a selected area of the ischemic region (Figure S3-A). In the area fraction method, we measured hypo-signals through the area fraction of T2*-weighted images. Using this method, we calculate the percentage of pixels in the image or selection that have been highlighted in the ischemic region (Figure S3-B).


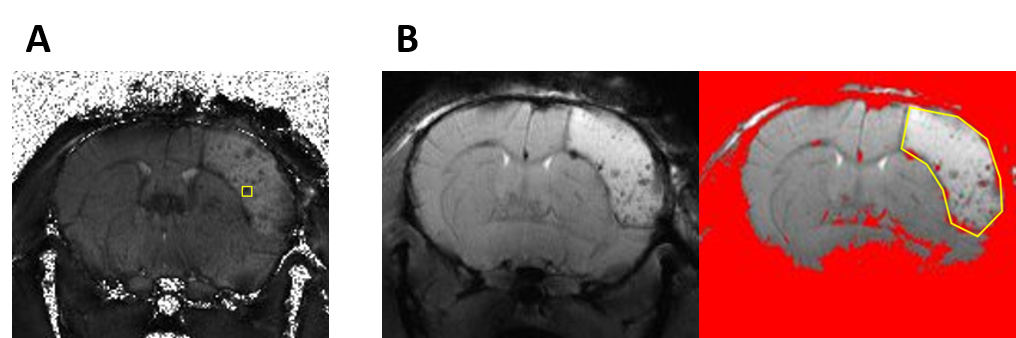


**Figure S8**. Methods to quantify the hypo-signals in T2*-weighted images. (A) Mean grey value using the maps of T2*-weighted images. The measured region is indicated in yellow. (B) Area fraction method using the T2*-weighted images. The ischemic area is signaled (yellow line).
